# A comprehensive, multi-center, immunogenomic analysis of melanoma brain metastases

**DOI:** 10.1186/s40478-025-02035-7

**Published:** 2025-06-02

**Authors:** Lucy Boyce Kennedy, Amanda E. D. Van Swearingen, Marissa R. Lee, Layne W. Rogers, Alexander B. Sibley, Jeff Sheng, Dadong Zhang, Xiaodi Qin, Eric S. Lipp, Swaminathan Kumar, Aron Joon, Pixu Shi, Michael A. Davies, Kouros Owzar, Carey K. Anders, April K.S. Salama

**Affiliations:** 1 Duke Cancer Institute, Duke University Medical Center, Durham, NC USA; 2 Duke Center for Brain and Spine Metastasis, Duke University Medical Center, Durham, NC USA; 3 Department of Biostatistics and Bioinformatics, Duke University School of Medicine, Durham, NC USA; 4 Department of Melanoma Medical Oncology, University of Texas MD Anderson Cancer Center, Houston, TX USA; 5 Department of Neurosurgery, Duke University School of Medicine, Durham, NC USA; 6 The Department of Hematology and Medical Oncology, Cleveland Clinic, 9500 Euclid Avenue CA-60, Cleveland, OH 44195 USA

**Keywords:** Melanoma, Brain metastases, Immune microenvironment, Macrophages, Autophagy

## Abstract

**Background:**

Melanoma brain metastases (MBM) have a unique molecular profile compared to extracranial metastases (ECM). Description of the biological features and clinical outcomes of MBM will facilitate the design of rational therapies.

**Methods:**

We examined the mutational landscape and gene expression profiles of MBM (74 patients) and ECM (34 patients) in paired patient samples from a previously published dataset with whole-exome sequencing (WES) and RNA sequencing (RNAseq) data from MD Anderson Cancer Center (MDACC). We also present findings from MBM from a new cohort of 14 patients from Duke University to strengthen investigation of somatic mutations and gene expression profiles. Gene Set Enrichment Analysis (GSEA) was used to compare paired MBM versus lymph node (LN) metastases and skin metastases. Relative immune cell abundance was inferred using deconvolution methods. Survival outcomes from craniotomy and associations with biological features, *BRAF* mutation status, and PTEN expression were assessed.

**Results:**

GSEA found that autophagy signaling pathways are enriched in MBM versus LN and skin metastases. *BRAF* was the most frequently mutated clinically relevant gene in MBM and ECM, with *NRAS* and *PTEN* also frequently altered in MBM. The most strongly upregulated genes in autophagy pathways were glial fibrillary acidic protein (*GFAP*) and hemoglobin beta (*HBB*). An increased proportion of immune-suppressive M2 compared to tumor-suppressive M1 macrophages in MBM and ECM was identified. There was not sufficient evidence for an association between *BRAF* V600 mutation status or expression and overall survival (OS) from craniotomy.

**Conclusions:**

The mutational landscape and gene expression of MBM from the Duke cohort resembled those previously reported in the MDACC cohort. Upregulation of autophagy pathways was observed in patient-matched MBM versus LN and skin metastases due to upregulation of two genes, *GFAP* and *HBB*. In MBM, higher M2:M1 ratio may contribute to a therapeutically relevant immune-suppressive tumor microenvironment (TME).

**Supplementary Information:**

The online version contains supplementary material available at 10.1186/s40478-025-02035-7.

## Introduction

Melanoma is the most aggressive type of skin cancer, and its incidence is increasing worldwide. It is estimated that, in the United States in 2025, there will be approximately 104,960 new diagnoses of and 8,430 deaths from cutaneous melanoma [57]. Melanoma exhibits a high degree of cerebral tropism, and up to 70% of patients with metastatic melanoma have central nervous system (CNS) metastases at the time of death [7]. Historically, the development of melanoma brain metastases (MBM) has been associated with a poor prognosis, with a median overall survival (OS) of approximately four months in retrospective series [55].

Recent advances in systemic therapies, including immune and targeted therapies, have improved the prognosis of advanced melanoma [8, 25, 67]. These agents have shown similar intracranial and extracranial efficacy in clinical trials of selected patients with MBM [13, 40, 60]. However, the pathogenesis of MBM remains poorly understood, and morbidity and mortality are significant. There remains an unmet need to develop novel treatment strategies for this patient population.

The literature describing the genetic landscape of brain metastases compared to extracranial metastases (ECM) in melanoma is limited, in part due to limited available brain metastasis tissue. Brastianos et al. described a pattern of branched evolution in which primary and metastatic tumors continued to evolve independently from a common ancestor, though this included only three patients with melanoma [5]. This finding of branched evolution highlights the importance of analyzing patient-matched intracranial and extracranial specimens to account for inter-patient heterogeneity, although available literature describing patient-matched MBM and ECM is limited, partly due to the challenges of obtaining the tissue specimens. On an individual patient level, focused hotspot sequencing of patient-matched MBM and ECM has shown concordance in the presence or absence of driver mutations [10].

The largest published cohort of patient-matched MBM and ECM identified heterogeneity of immune cell infiltration in MBM and found that increased infiltration of immune cell types including T cells, natural killer (NK) cells, and monocytic lineage cells, was associated with increased OS from craniotomy [18]. A recent gene expression profiling study of patient-matched intracranial and extracranial metastases from 16 unique patients identified enrichment of calcium signaling pathway genes, down-regulation of genes involved in immune signaling pathways, and presence of a brain-like gene expression pattern in MBM compared to ECM [35]. Patient-specific clustering was observed, demonstrating the importance of studies comparing patient-matched intracranial and extracranial metastases.

Multiple studies have identified differences in immune cell populations and immune signaling in MBM compared to ECM. A cohort study which included a small cohort of single-cell RNA sequencing (scRNAseq) data from resected MBM demonstrated a unique microenvironment compartment with decreased macrophage and T cell populations in MBM with *BRAF* V600E mutation compared to MBM without *BRAF* V600E mutation, suggesting that genetic factors may influence the microenvironment [64]. Further, multiple studies have demonstrated enrichment in oxidative phosphorylation (OXPHOS) in MBM compared to ECM [18, 29]. This finding is clinically targetable and suggests that metabolic derangements may alter the tumor microenvironment (TME), promoting tumor growth.

This study sought to investigate alterations in mutational composition and gene expression profiles in MBM compared to ECM and to characterize the abundance of immune cell subpopulations. We performed an analysis of a previously published dataset consisting of DNA whole-exome sequencing (WES) of 44 samples from 17 patients with matched blood samples and RNA sequencing (RNAseq) of 138 samples from 80 patients from MD Anderson Cancer Center (MDACC) [18], as well as reporting some select relevant findings from two separate MDACC datasets consisting of: (1) *n* = 54 primary cutaneous melanoma (PCM) samples with known recurrence status (recurrence in brain (MBM), extracranially (ECM), or none (no recur)) [21, 36] and (2) four different xenograft models implanted intracranially (ICr) versus subcutaneously (SQ) [18]. We also generated and analyzed an independent dataset of 14 newly-sequenced resected MBM from 14 patients from Duke University Medical Center using WES and RNAseq to describe the genetic and immune landscape of MBM. We identified enrichment of autophagy signaling pathways in MBM versus lymph node (LN) and skin metastases, which was driven by *GFAP* and *HBB* expression. Immune cell analysis identified an increased proportion of immune-suppressive M2 compared to tumor-suppressive M1 macrophages in MBM and ECM. Finally, we assessed for association between *BRAF* V600 mutation and *PTEN* expression and survival from craniotomy. This analysis provides further insight into the pathogenesis and TME of MBM.

## Materials and methods

### Patient cohort

**Fig. 1:**
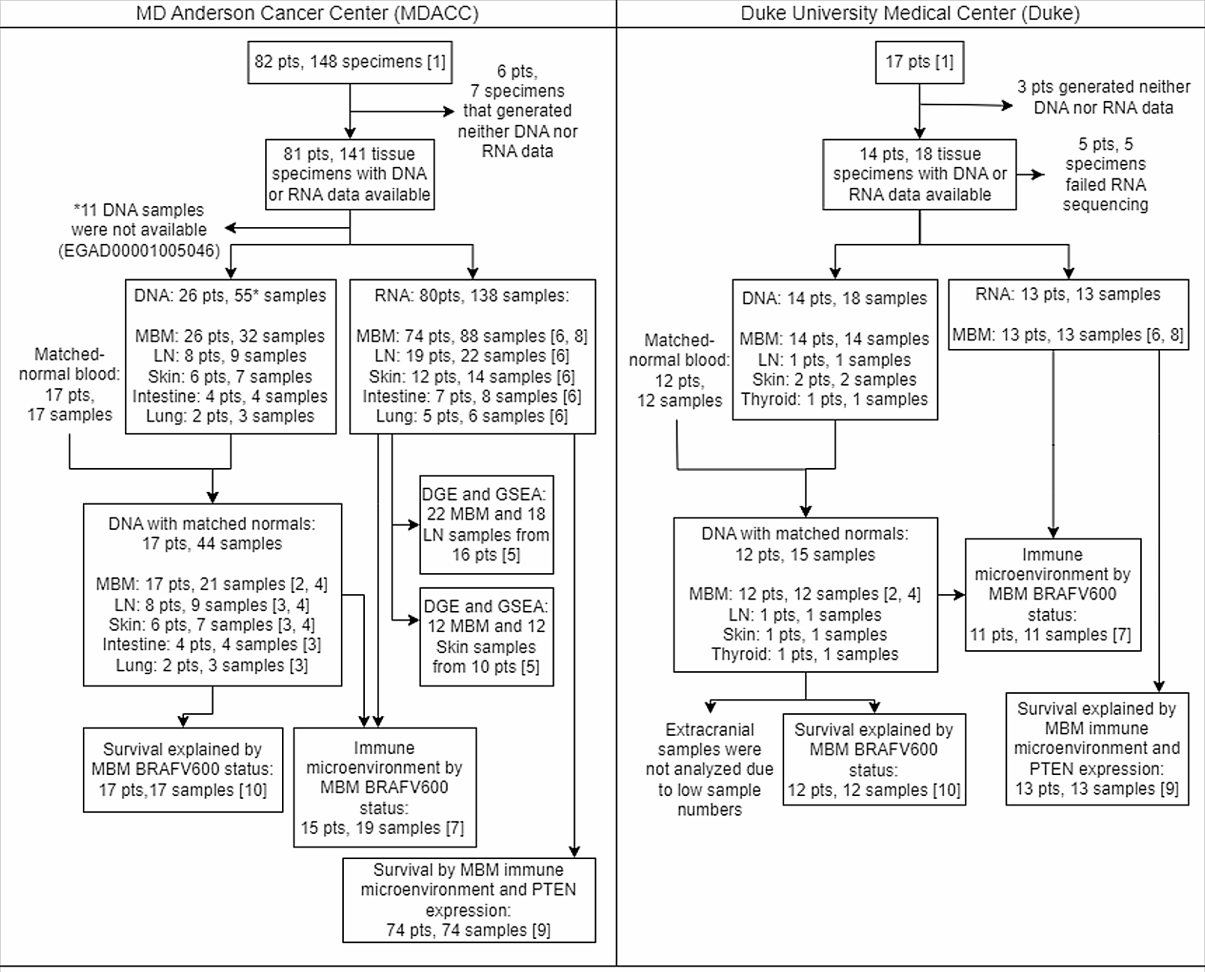
Consort diagram of MDACC and Duke dataset patients represented in RNA and DNA samples analyzed. The MDACC dataset is previously published [18]. Analyses utilizing these cohorts: [1] Overall survival by cohort (Fig. 2) [2], Somatic mutations in MBM (Fig. 3) [3], Somatic mutations in ECM (Fig. 4) [4], High-frequency somatic alterations (Fig. 5), top somatic alterations by dN/dS analysis (Fig. 6), and shared mutations between cohorts and tumor locations (Fig. 7) [5], DGE and GSEA comparing MBM vs. LN and MBM vs. skin metastases (Fig. 8) [6], Tumor immune microenvironment by cohort and tumor location (Fig. 9) [7], Tumor immune microenvironment in MBM by *BRAF* V600 status (Fig. 10a, b) [8], Tumor immune microenvironment in MBM by *PTEN* expression (Fig. 10c, d) [9], Survival by immune microenvironment in MBM (Fig. 11a, b), by *PTEN* expression in MBM (Fig. 12c, d) [10], Survival by *BRAF* V600 status in MBM (Fig. 12a, b)

A previously published dataset (available from EGA: EGAD00001005046) from MDACC based on 82 patients was obtained through a data transfer agreement. It includes RNAseq on surgically resected, FFPE MBM (88 tumor samples (m) from 74 patients (n)) and surgically resected ECM from an overlapping set of patients (hereafter “MDACC dataset”, Fig. 1) [18]. WES data was available from tumor samples with accompanying matched blood samples for MBM (*n* = 17, m = 44) and ECM from an overlapping set of patients (Fig. 1). A second previously published dataset from MDACC was available on 54 primary cutaneous melanoma (PCM) samples from 54 patients, including RNAseq from 19 PCM which did not recur, 19 PCM which recurred as MBM, and 16 PCM which recurred as ECM [21, 36] (hereafter “PCM dataset”). Methods for sample selection, preparation, library construction, and sequencing of MBM, ECM, and PCM in this patient cohort have been previously published [21, 36].

For the independent cohort, 17 patients with MBM who underwent craniotomy between 1988 and 2017 at Duke University Medical Center and had banked MBM specimens were identified (hereafter “Duke dataset”, Fig. 1). Of those, 14 patients with advanced melanoma (13 cutaneous, one acral) had available frozen MBM tissue that generated RNA or DNA sequencing data (Fig. 1). Tissue was obtained from the Duke Brain Tumor Center Biorepository (BTBR, IRB Pro00007434) at Duke University. The Duke University BioRepository and Precision Pathology Center (BRPC) and Surgical Pathology archives were searched for patient-matched extracranial tumor tissue. Extracranial tumor samples (*n* = 4) were FFPE tissue blocks (Fig. 1). Due to the small number of Duke extracranial tumor samples, these samples ultimately were not included in our analyses. Collection of tissue samples and clinical information was approved by the Duke University Institutional Review Board (Pro00103267).

### Animal xenograft model

Five human melanoma cell lines, A375, A375R1, MEL624, MEWO, and WM1361A, were established, harvested, and processed in CD-1 nude mice as either intracranial (ICr) or subcutaneous (SQ) tumors, as described in Fischer et al. [18] (hereafter “Xenograft dataset”). A375, MEWO, and WM1361 were established from primary tumors. A375-R1 is a MEK inhibitor-resistant subclone of A375 [20]. The tumor site/source of MEL624 is unknown. The sample set includes three ICr and three SQ tumor samples per cell line, except for the A375R1 cell line which had two ICr samples. Due to insufficient ICr samples, the A375R1 cell line was excluded from downstream analyses.

### Tissue sample preparation of Duke dataset samples

H&E stained slides were prepared from patient-derived, frozen MBM tissue. Slides were reviewed by a pathologist, and regions containing at least 50% viable tumor were selected to guide macrodissection of the tissue block.

### RNAseq of Duke dataset samples

Extraction of RNA from MBM, which were frozen, was performed using the RNeasy^®^ Plus Mini Kit according to the manufacturer’s protocol (QIAGEN, CAT #74134, Germantown, MD).

Extracted total RNA quality and concentration were assessed on a 2100 Bioanalyzer (Agilent Technologies) and Qubit 2.0 (ThermoFisher Scientific). RNAseq libraries were prepared using the Illumina Stranded Total RNA Prep with Ribo-Zero Plus kit (Illumina, CAT #20040529) and indexed using Illumina RNA UD Indexes Set A (Illumina, CAT #20040553). All libraries were then pooled in equimolar ratio and sequenced on 1 lane of an Illumina NovaSeq 6000 S4 flow cell, generating 150 bp PE reads. Sequence data were demultiplexed and Fastq files generated using Illumina Bcl2Fastq conversion software.

### Whole-exome sequencing of Duke dataset samples

Extraction of DNA from the isolated tissue was performed using the Gentra^®^ Puregene^®^ Tissue Kit (QIAGEN, CAT # 158066, Germantown, MD) and extraction of DNA from blood was performed using the Gentra Puregene Blood Kit (QIAGEN, CAT #158026), both according to the manufacturer’s protocols.

Extracted DNA was quantified using Qubit (Thermo Fisher Scientific). DNA-seq libraries were prepared for each sample using the Kapa Hyper Prep kit (Roche, Cat #KK8504) and indexed using IDT for Illumina DNA unique dual indexes (UDI) Set A. Final libraries were quality checked using 2100 Bioanalyzer (Agilent Technologies) and Qubit 2.0 (ThermoFisher Scientific) and pooled in batches of 12 (pre-capture pooling). Each pool of 12 libraries were then hybridized with IDT Human xGen Exome Research Panel V1 probes (IDT, CAT #1056114) to capture and pull down the portion of the DNA-seq library representing the human the exome. Final captured libraries were amplified, pooled and sequenced using 2 lanes of an Illumina NovaSeq 6000 S4 flow cell, generating 150 bp PE reads. Sequence data was demultiplexed and Fastq files generated using Illumina Bcl2Fastq conversion software.

### Analysis of MDACC and Duke whole-exome sequencing data

WES data were processed and analyzed through the following procedures. First, raw sequences were mapped to the hg38 reference genome using the BWA-MEM v0.7.17 algorithm [37]. The reference genome was obtained from the publicly available GATK resource bundle v0 (https://gatk.broadinstitute.org/hc/en-us/articles/360035890811-Resource-bundle). Then, aligned BAM files were preprocessed using Picard v3.0.0 and GATK v4.4.0.0 [14, 45, 63] to remove duplicate reads and perform base recalibration.

Somatic variant calling was performed by first constructing a panel of normals VCF of common artifactual and germline variant sites using all normal tissue samples (GATK v4.4.0.0 Mutect2, GenomicsDBImport, CreateSomaticPanelOfNormals). Somatic variants were then called in all tumor samples using GATK v4.4.0.0 Mutect2 with default parameters and an additional flag (--f1r2-tar-gz) for collecting F1R2 counts as input for LearnReadOrientationModel. When available, patient-matched normal tissue controls (--input flag for normal BAM file and–normal-sample for specifying the normal library name) were used in combination with the panel of normals VCF (--panel-of-normals) to call somatic variants in tumor samples with Mutect2. For patients lacking a matched normal tissue sample, only the panel of normals VCF was used to call somatic variants in tumor samples with Mutect2. Initial callsets were prepared for filtering by first estimating the fraction of reads introduced by cross-sample contamination (GATK v4.4.0.0 GetPileupSummaries, CalculateContamination), and second, using the F1R2 counts collected by Mutect2 to calculate prior probabilities of single-stranded substitution errors prior to sequencing for each trinucleotide context (GATK v4.4.0.0 LearnReadOrientationModel). Finally, callsets were filtered using GATK v4.4.0.0 FilterMutectCalls with default thresholds. Filtered callsets (single nucleotide variants (SNVs) and insertion/deletions (indels)) were then annotated using Ensembl Variant Effect Predictor (VEP) v110.1 [46] and converted to MAF format using vcf2maf v1.6.21 [33]. The maftools [44] R package was then used to visualize and summarize somatic variant types across samples.

The previously published MDACC dataset included a survey of detected mutations within 73 clinically relevant genes [16]. For this study, oncoprint plots of these clinically relevant genes were generated for MDACC MBM, Duke MBM, and MDACC ECM. Oncoprint plots for the top 10 genes with the highest frequency of somatic alterations were also generated for MDACC MBM, Duke MBM, MDACC LN, and MDACC skin samples. To evaluate concordance of the ranks of gene mutation frequencies between sample sets (e.g., MDACC MBM versus Duke MBM, MDACC LN, and MDACC Skin), the Kendall rank correlation coefficient (Tau) [26] was used. Concordance of gene mutation frequency between two sample sets was determined if Tau significantly differed from zero with type I error probability controlled at 0.05. To account for the influence of many shared, low-frequency mutations across sample sets, correlations in the full list of genes and genes with a mutation frequency of at least 50% were evaluated for each sample set comparison.

To identify high frequency somatic alterations corrected for baseline synonymous mutations among MDACC MBM, Duke MBM, MDACC LN, and MDACC skin samples, we used the dNdScv (v0.1.0) R package [43] to perform gene-level neutrality tests by estimating dN/dS ratios for missense, nonsense, indel, and essential splice site mutations. The dNdScv method was run for MDACC MBM, Duke MBM, MDACC LN, and MDACC skin sample groups by pooling together somatic mutation calls across samples within each group. Default dNdScv parameters were used except reference files were configured for the hg38 reference genome, `cv` was set to NULL to avoid using the default covariate matrix built for hg19, and `max_coding_muts_per_sample` was set to Inf to retain all samples due to limited sample sizes and no clear outliers in total coding mutations. Genes were ranked by the unadjusted global P value (pglobal_cv), representing the probability of non-neutral selection based on all mutation types and those with a global q-value (qglobal_cv) < 0.1 adjusted for multiple comparisons were considered significant.

### Analysis of MDACC and Duke RNA sequencing data

RNAseq data from MDACC and Duke dataset samples were analyzed through the following procedures. The quality of the raw sequencing reads was evaluated and reported using FastQC v0.11.8 and MultiQC v1.9 [2, 16]. Sequences with adapter contamination and low-quality sequences were cleaned using Trimmomatic v0.39 [4]. The quality of the remaining sequences was reevaluated to guarantee minimum adapter contamination.

The raw sequencing reads were aligned to the reference genome using the STAR aligner v2.7.2b [15]. The aligned reads were then mapped to annotated genomic features, including genes and exons, using STAR’s built-in module. The human reference sequence (GRCh38.p12) and annotation GTF file (hg38) were obtained from GENCODE [23, 24]. Mapping quality was evaluated before any downstream analyses. The read level mapping quality was evaluated through STAR output, including the fraction of reads mapped to gene regions, ambiguous regions, non-feature regions or multiple loci. Likewise, the base level mapping quality was accessed through CollectRnaSeqMetrics from Picard Toolkits v2.20.7 [6].

Differential gene expression (DGE) in MDACC dataset samples were analyzed within the framework of a negative-binomial model using the R [53] extension package DESeq2 v1.38.3 [41]. Two contrasts were analyzed using data from the MDACC cohort: MBM versus skin or LN with MBM set as the baseline level, where samples from the same patient were pair-matched. False-discovery-rate (FDR)-adjusted p-values were reported. Additionally, the lfcshrink function of the apeglm v1.27.0 package [73] was used to generate Log_2_ fold change shrinkage estimates for downstream analyses. Log_2_ fold changes greater than 1 or less than − 1 with an adjusted p-value < 0.05 were considered significant. Gene set enrichment analysis (GSEA) was conducted on the same set of contrasting groups to determine whether gene sets in the pathway database Reactome [12, 17] were enriched using the R extension package ReactomePA v1.42.0 [71]. Default parameters were used except for pvalueCutoff which was set to 0.25 to retain additional pathway results, and number of permutation replicates (nPerm) which was set to 100,000. This package was also used to visualize gene expression across the gene network for select gene sets.

To investigate immune cell infiltration in MDACC MBM and ECM (intestine, LN, lung, skin) and Duke MBM, we used the CIBERSORTxFractions module within the CIBERSORTx software [49] to infer the relative fraction of 22 immune cell types based on gene expression data and the LM22 [49] signature matrix. We followed the CIBERSORTx developers’ recommendation to analyze the Duke and MDACC datasets separately since there are expected batch effects [59]. To visualize differences in immune cell fractions between (i) MBM by cohort, (ii) MBM and ECM sites (intestine, LN, lung, skin) in the MDACC cohort, (iii) MBM samples from patients with the presence/absence of a *BRAF* V600 mutation in the MDACC and Duke cohorts, and (iv) MBM samples with low/high *PTEN* expression in MDACC and Duke cohorts, dotplots, boxplots, and violin plots were used. *PTEN* expression levels were dichotomized using the median variance-stabilized expression value transformed by the package DESeq2 [41].

To compare the ratio of M2 to M1 macrophages between cohorts and sites, cell fraction estimates were first transformed using the equation atan(log(M2/M1)/pi * 2)) to account for the compositional nature of the data and the presence of zeros. Samples with zero M1 and M2 fractions were excluded from analyses. Then, Wilcoxon rank-sum tests were used to compare sample groups (MDACC MBM, MDACC LN, MDACC Skin, and Duke MBM) and the resulting p-values were adjusted using the Bonferroni method to account for multiple testing.

### Survival analysis

Cox proportional hazards (PH) regression was used to perform time-to-event analyses on the time from craniotomy to death or last follow-up using the R extension package survival v3.5.5 [61]. The previously published MDACC dataset included sample-level survival times as months from accession. To perform patient-level survival analyses for the MDACC cohort, we selected the RNA or DNA sample with the maximum number of months from accession to death or last follow-up (i.e., earliest craniotomy) for each patient. If a patient had multiple samples extracted on the same date, one sample was selected at random. For univariate Cox PH models, hazard ratios (HR), 95% confidence intervals (CI), and p-values from the score test are reported. For multivariate Cox PH models, p-values from Wald tests were used instead. The previously published analysis of the MDACC dataset reported that increased T cells, CD8 + T cells, cytotoxic lymphocytes, NK cells, and monocytic lineage cells were each associated with improved OS from craniotomy [18]. Therefore, four Cox PH models were fitted, with the first comparing survival between MDACC and Duke cohorts, the second evaluating the effect of infiltrating immune cell fractions stratified by cohorts, the third evaluating the effect of *BRAF* V600 mutation status within cohorts, and the fourth evaluating the effect of *PTEN* expression within cohorts.

For the effect of immune cell infiltration on survival, the fractions of M1 and M2 macrophages and CD4+, and CD8 + T cells were divided by the total fraction of other cell types and log-transformed before being used as predictors to account for the characteristics of compositional data. The CD4 + fraction consisted of the sum of all three CD4 + cell types (i.e. naïve, memory resting, and memory activated). For the effect of *PTEN* expression on OS, RNA expression values were variance-stabilizing transformed by the R package DESeq2 v1.38.3 [41] before being used as a predictor. Kaplan-Meier (KM) plots were generated to illustrate selected associations with time to event outcomes. For the purposes of illustrating associations with continuous variables, covariates were dichotomized at the median.

### Analysis of PCM and xenograft RNA sequencing data

RNAseq data for PCM samples preprocessed as described in Kwong et al. [36], and ICr and SQ xenograft samples preprocessed using the Xenome pipeline [11] as described in Fischer et al. [18]. The resulting gene-level count data were received from MDACC.

DGE analyses in the PCM and xenograft datasets were performed within the framework of a negative-binomial model using the R [53] extension package DESeq2 v1.38.3 [41]. Accordingly, the RSEM expected counts generated during preprocessing of these datasets were rounded to the nearest integer. In addition, features were removed if they had Entrez IDs that did not map to HGNC-approved gene symbols that were obtained on March 25, 2025 using the genenames.org custom download tool [56]. Four contrasts were evaluated for the PCM dataset: (i) PCM which recurred as ECM (m = 16) versus PCM which did not recur (m = 19), (ii) PCM which recurred as MBM (m = 19) versus PCM which recurred as ECM (m = 16), (iii) PCM which recurred as MBM (m = 19) versus PCM which did not recur (m = 19), and (iv) any recurrence (MBM and ECM; m = 35) versus PCM which did not recur (m = 19). One contrast was evaluated for the xenograft dataset within each cell line: ICr versus SQ. The second group listed in the contrasts was treated as the reference group.

For the downstream gene-level differential expression analyses we used the same methods as for the MDACC and Duke datasets. To determine whether the Autophagy pathway (R-HSA-9612973) in the Reactome database [12, 17] was enriched for the same set of contrasts, GSEA was conducted using the R extension package ReactomePA v1.42.0 [71]. Default parameters were used except for scoreType which was set to “pos” to perform a one-sided test to identify up-regulated pathways, pvalueCutoff which was set to 1 to retain all pathway results, and permutation replicates (nPerm) was set to 100,000.

## Results

### Clinical characteristics of the UT MD Anderson and Duke melanoma craniotomy cohorts

To better characterize the molecular profile of MBM, we re-examined the mutational landscape and gene expression profiles in patient samples from a previously published cohort with WES and RNAseq data generated for patient-matched MBM and ECM from MDACC [18]. To expand this analysis, we conducted analyses in an independent cohort of MBM from Duke University Medical Center. Seventeen patients with MBM who underwent craniotomy between 1988 and 2017 and had banked MBM specimens were included in the Duke cohort (Fig. 1). Of those, MBM specimens from 14 unique patients yielded sequencing results. WES data was available from tumor samples with accompanying matched blood samples for 12 MBM from 12 unique patients, with patient-matched extracranial tumors available for three unique patients, including one lymph node (LN), one primary cutaneous melanoma, and one thyroid sample (Fig. 1). RNAseq data was available on 13 MBM from 13 unique patients (Fig. 1). No patient-matched extracranial tumors yielded RNAseq data. Due to the low number of matched extracranial tumor samples and lack of RNAseq data available in the Duke cohort, these samples were not included in the analyses. MBM from one patient had only WES (without RNAseq) data due to poor RNAseq library quality. Specimens included in our analyses of the MDACC and Duke datasets are summarized in Fig. 1.

Clinical details of patients included in the Duke cohort are summarized in Table 1. 13 of 14 patients that yielded sequencing results underwent craniotomy between 2003 and 2017; the remaining patient underwent craniotomy in 1988. Four patients were known to have received systemic therapy for melanoma prior to craniotomy, and six patients were recorded as having received systemic therapy after craniotomy (Table 1). Three patients received stereotactic radiosurgery for MBM prior to craniotomy, and nine patients received either stereotactic radiosurgery (SRS), whole brain radiotherapy (WBRT), or both after craniotomy. Six patients were diagnosed with MBM as their initial melanoma diagnosis without evidence of extracranial disease and without a known primary melanoma. The clinical characteristics of patients included in the MDACC dataset were previously published [18].

**Table 1:** Duke dataset cohort characteristics. Clinical and molecular characteristics of the Duke MBM dataset cohort for patients with WES and/or RNAseq data available

Sex	*n* = 14 pts
Male	11
Female	3
**Age**	**Median**,** range (years)**
At craniotomy	62 (45,84)
**Race**	***n*** ** = 14 pts**
Caucasian	14
**Melanoma type**	***n*** ** = 14 pts**
Cutaneous	13
Acral	1
**Location of primary melanoma**	***n*** ** = 14 pts**
Head and neck	2
Extremity	3
Trunk (multiple tumors)	1
Unknown	7
No information	1
**Stage at diagnosis of primary melanoma**	***n*** ** = 14 pts**
IIA	2
IIC	1
Unknown primary	7
No information about primary	4
**Brain metastases present at first diagnosis of metastatic disease**	***n*** ** = 14 pts**
Yes	7
No	6
Unknown	1
**Clinical Molecular Testing and Resulting Molecular Characteristics**	***n*** = 14 pts*
Not tested	4
No information available	1
Test unknown	1
BRAF V600E	1
Targeted mutation analysis	6
BRAF V600 mutation analysis	3
BRAF V600E	1
BRAF V600K	1
BRAF V600 mutation negative	1
BRAF mutation analysis	2
BRAF mutation negative	2
NRAS mutation analysis	1
NRAS Q61	1
KIT mutation analysis	3
KIT mutation negative	3
Melanoma hotspot	1
No actionable mutations	1
Foundation One	1
NF1 W696*	1
ROS1 S941F	1
TET2R544*	1
CDKN2A mutation	1
TP53 mutation	1
LRP1B mutation	1
TERT promoter mutation	1
**Systemic therapy**	*n* = 14 pts*
Before craniotomy	
Pembrolizumab	1
Ipilimumab-nivolumab	1
High-dose IL-2	3
Biochemotherapy	1
Interferon	1
Isolated limb infusion	1
None	9
No information	1
After craniotomy	
Pembrolizumab	1
Nivolumab	1
Ipilimumab	1
Ipilimumab-nivolumab	2
Dabrafenib	1
Trametinib	1
Temozolomide	2
None	4
Unknown	3
No information	1
**Radiation therapy**	*n* = 14 pts
SRS	6
WBRT	1
SRS and WBRT	2
None	1
None pre-craniotomy; post-craniotomy RT not known	3
No information	1
**Timing of CNS radiation**	
After craniotomy	6
Both before and after craniotomy	3
None	1
Unknown	3
No information	1

*Due to some patients having multiple molecular tests/mutation results or systemic therapies, patient tallies in these sections do not total and will exceed 14 patients

**Fig. 2:**
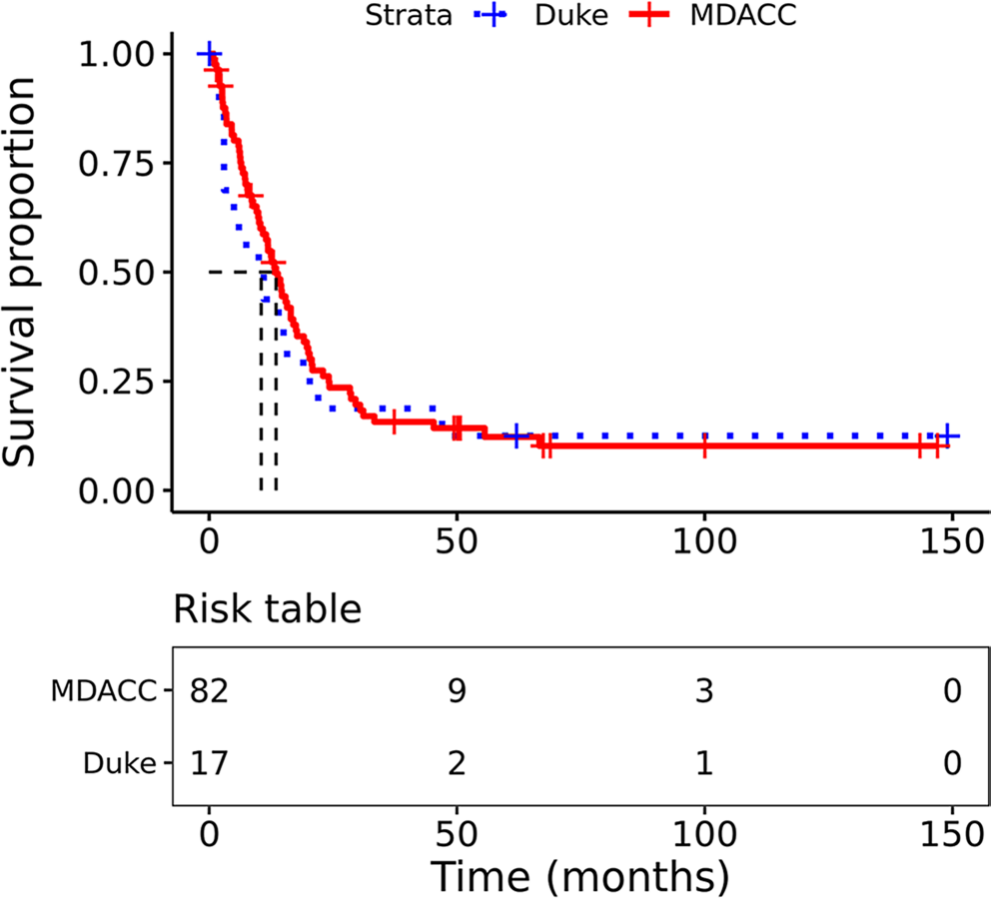
Overall survival of the MBM cohorts. Kaplan-Meier OS analysis from craniotomy of patients in the MDACC (red) and Duke (blue) cohorts. Median survival and 95% CI for MDACC was 13.5 months (10.8–17.5) and for Duke was 10.5 months (3.0–47)

OS from craniotomy in the Duke and MDACC cohorts is shown in Fig. 2 (this included all patients for whom clinical data were available). There was no difference in OS from craniotomy comparing between the MDACC and Duke datasets (Cox PHmodel hazard ratio (HR) and 95% confidence interval (CI) = 0.864 (0.486–1.54), *p* = 0.617). Median OS from craniotomy and 95% CI for MDACC was 13.5 months (10.8–17.5) and for Duke was 10.5 months (3.0–47).

### Landscape of somatic mutations in MBM and ECM

**Fig. 3:**
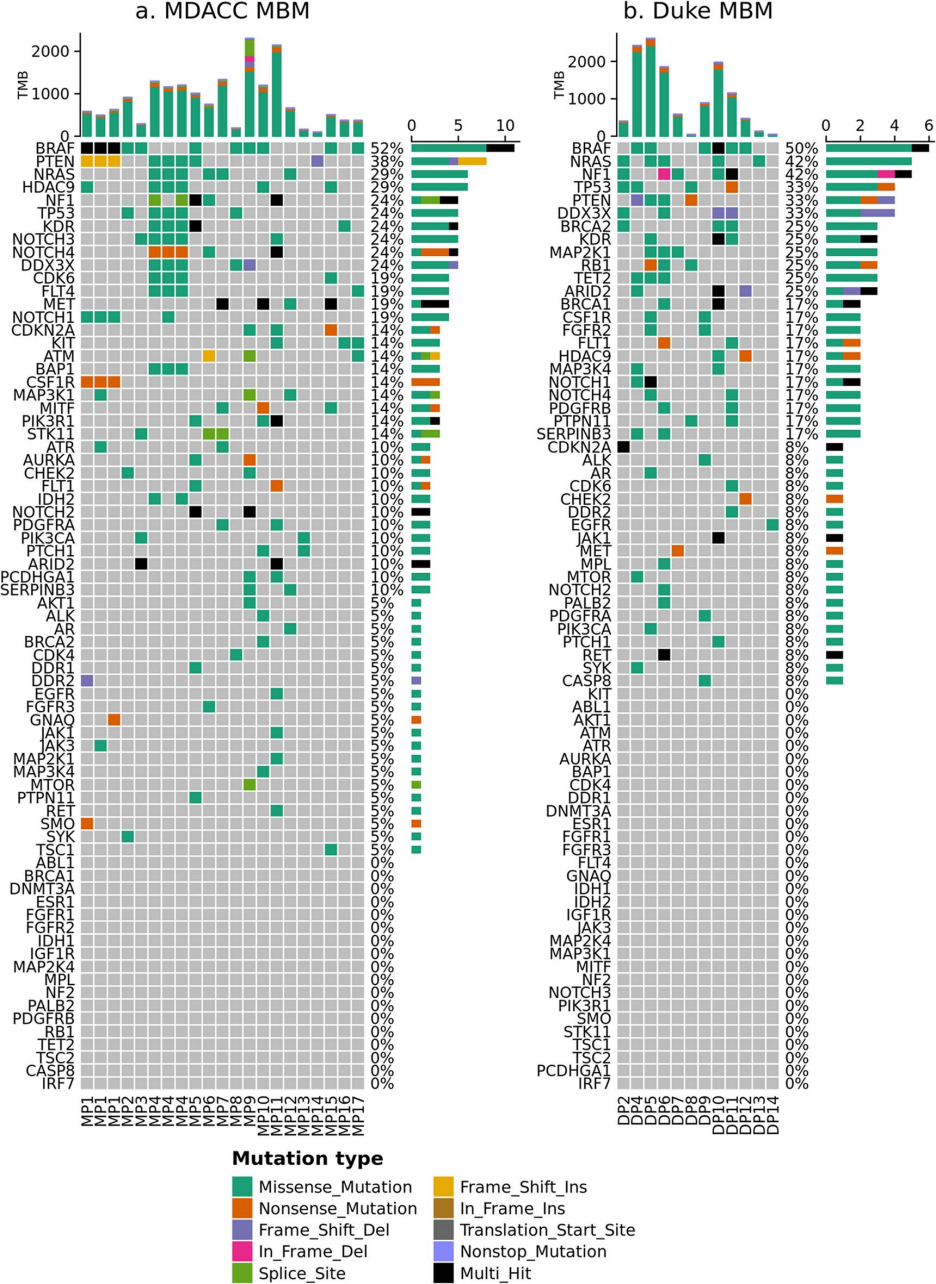
Somatic mutations in MBM. Oncoprint plots showing the frequency of non-synonymous somatic mutations in 73 clinically relevant genes in (**a**) MDACC MBM (*n* = 17, m = 21) and (**b**) Duke MBM (*n* = 12, m = 12). Right barchart shows the number of tumor samples with at least one non-silent mutation by mutation type; samples with more than one mutation type are indicated by “Multi_Hit”. Top barchart shows tumor mutational burden (TMB) as the number of non-silent mutations by mutation type called across the genome.

**Fig. 4:**
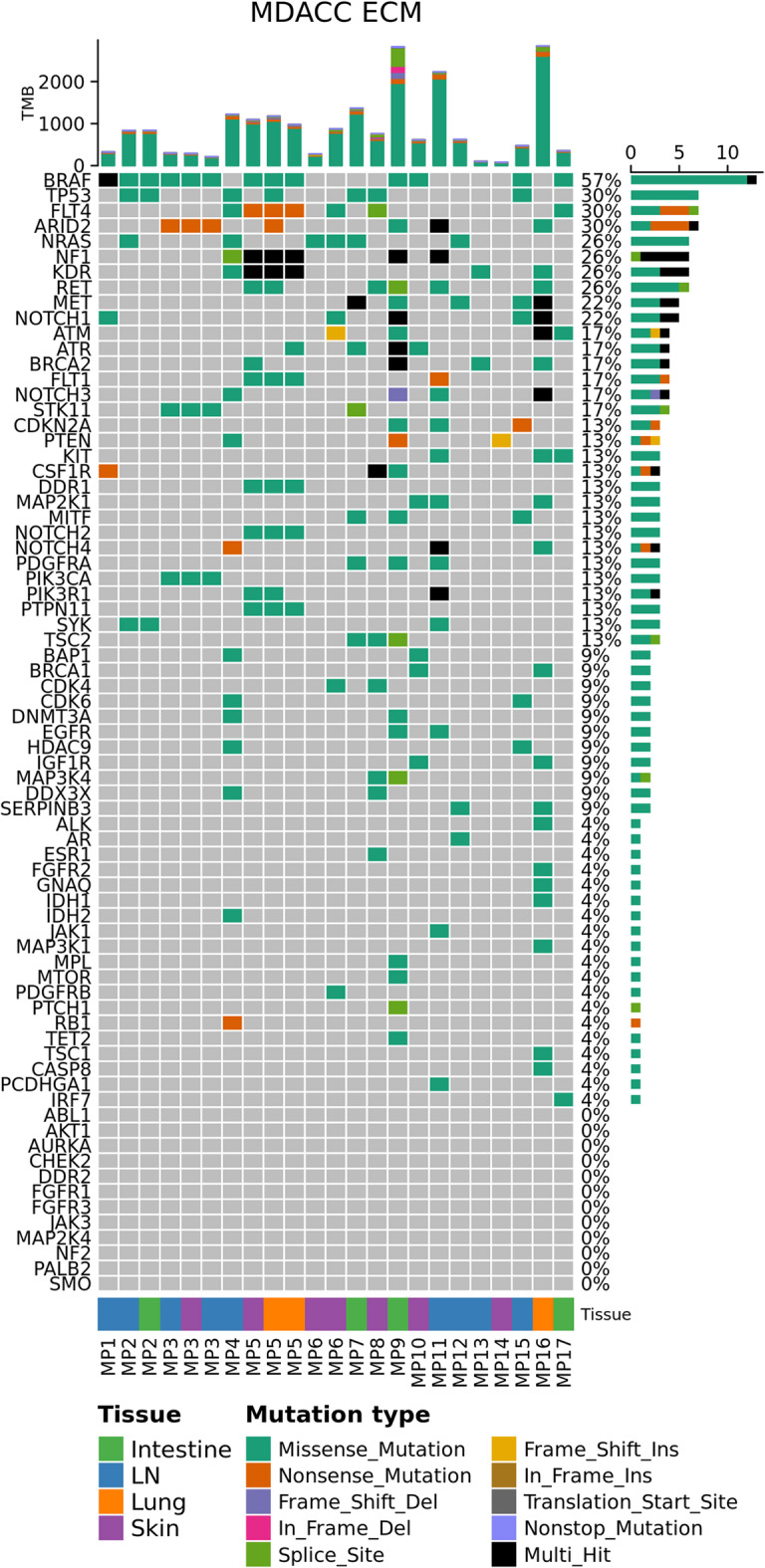
Somatic mutations in ECM from patients with MBM. Oncoprint plot showing the frequency of non-synonymous somatic mutations in 73 clinically relevant genes in MDACC ECM (Intestine *n* = 4, m = 4; LN *n* = 8, m = 9; Lung *n* = 3, m = 2; Skin *n* = 6, m = 7). Right and top barcharts are as described in Fig. 3

First, we described the frequency of non-synonymous mutations in 73 clinically relevant genes using WES data from the MDACC (21 MBM and 23 ECM from 17 unique patients) and Duke (12 MBM from 12 patients) datasets (Figs. 3 and 4). The frequency of non-synonymous somatic mutations in the same 73 clinically relevant genes in the MDACC dataset has been previously published [18]; as previously reported, there was no significant difference in the number of genes with non-synonymous mutations, comparing between patient-matched MBM and ECM [18]. The most commonly mutated gene within the clinically relevant genes among Duke MBM, MDACC MBM and MDACC ECM was *BRAF*, with mutations occurring in 50%, 52%, and 57% of samples, respectively. Following *BRAF*, the five most common genes with non-synonymous mutations in MDACC MBM were phosphatase and tensin homolog (*PTEN*), neuroblastoma RAS viral oncogene homolog (*NRAS*), histone deacetylase 9 (*HDAC9*), Neurofibromin 1 (*NF1*), and *TP53*, with mutations occurring in 38%, 29%, 29%, 24%, and 24% of samples, respectively. Following *BRAF*, the five most common genes with non-synonymous mutations in Duke MBM were *NRAS*, *NF1*, *TP53*, *PTEN*, and DEAD-Box helicase 3 X-Linked (*DDX3X)*, with mutations occurring in 42%, 42%, 33%, 33%, and 33% of samples, respectively. Following *BRAF*, the five most common genes with non-synonymous mutations in MDACC ECM were *TP53*, *FLT4*, *ARID2*, *NRAS*, and *NF1*, with mutations occurring in 30%, 30%, 30%, 26%, and 26% of samples, respectively.

**Fig. 5:**
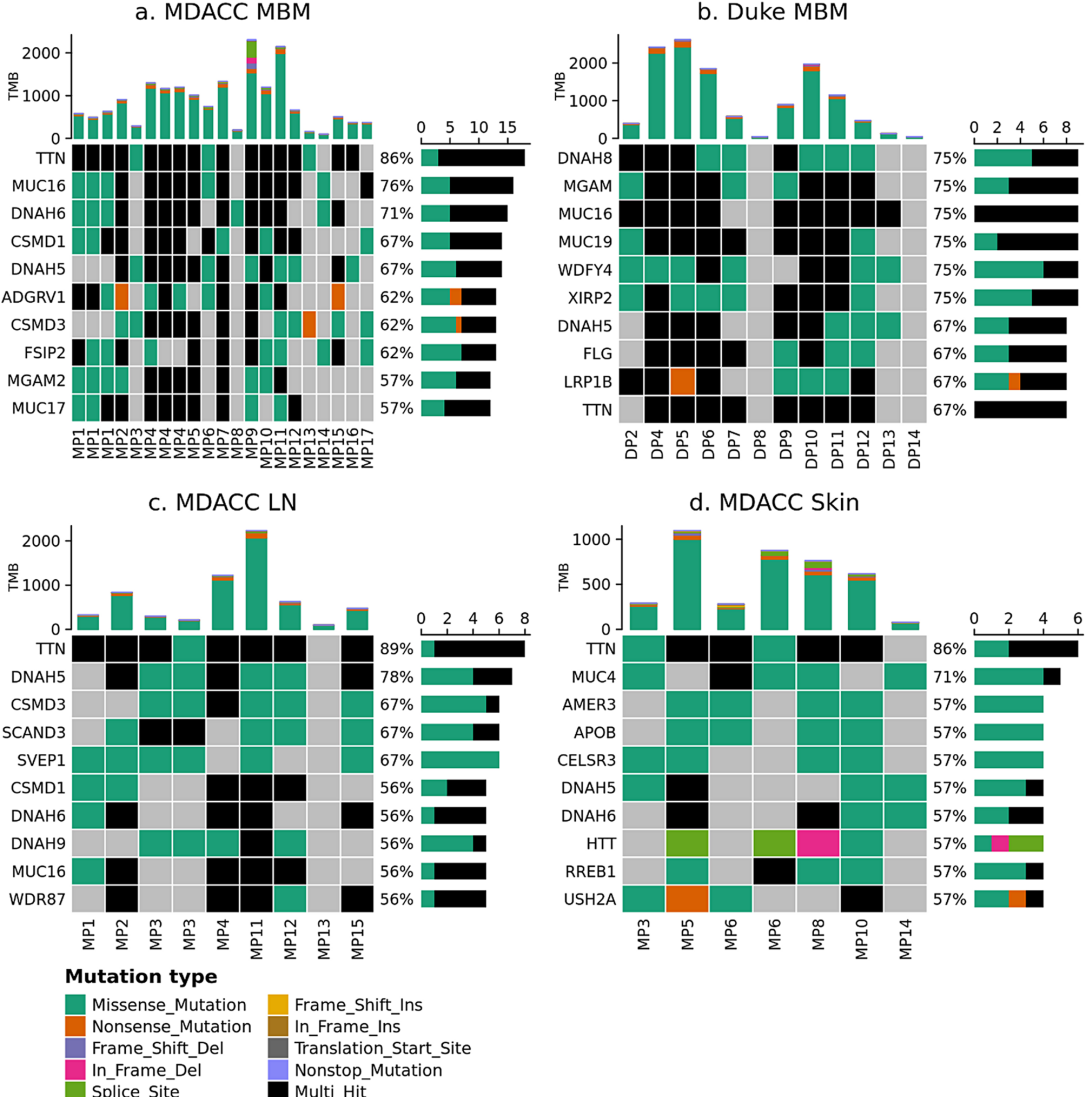
High frequency somatic alterations. Top ten genes with highest frequency of non-synonymous somatic alterations in (**a**) MDACC MBM (*n* = 17, m = 21), (**b**) Duke MBM (*n* = 12, m = 12), (**c**) MDACC LN metastases (*n* = 8, m = 9), and (**d**) MDACC Skin metastases (*n* = 6, m = 7). Right and top barcharts are as described in Fig. 3.

Next, we assessed the frequency of non-synonymous, stop-gain, and stop-loss mutations using WES data from brain, LN, and skin metastases in the MDACC dataset and MBM in the Duke dataset (Fig. 5). The landscape of genes with the highest frequency of somatic alterations was similar between brain, LN, and skin metastases. For example, comparing MBM, LN, and skin metastases in the MDACC dataset, titin (*TTN*) and Dynein axonemal heavy chain 5 (*DNAH5*) were included in the top 10 genes with the highest frequency of somatic alterations at all three sites. We also observed overlap between genes with high frequency of somatic alterations in the Duke cohort compared to the MDACC cohort. For instance, *TTN* and *DNAH5* were among the top 10 genes with highest frequency of somatic alteration in Duke MBM in addition to MDACC MBM, LN, and skin metastases. Somatic alterations in *TTN* were identified in 67%, 86%, 89%, and 86% of samples, respectively. *MUC16* was also among the top 10 most frequently mutated genes in both the MDACC and Duke MBM.

**Fig. 6:**
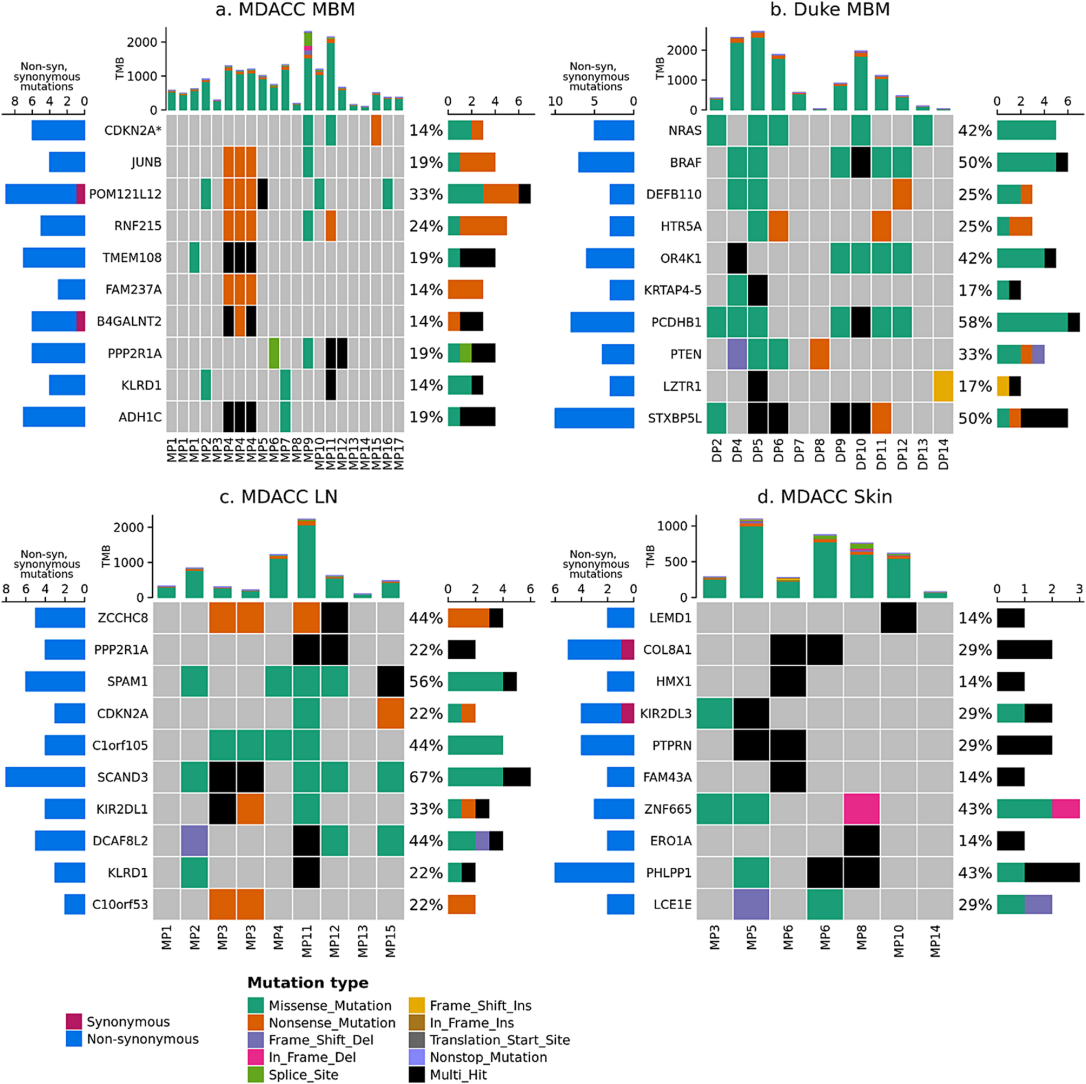
High frequency somatic alterations based on dN/dS analysis. Top ten genes sorted by dNdScv unadjusted global P value in (**a**) MDACC MBM (*n* = 17, m = 21), (**b**) Duke MBM (*n* = 12, m = 12), (**c**) MDACC LN metastases (*n* = 8, m = 9), and (**d**) MDACC Skin metastases (*n* = 6, m = 7). The dNdScv unadjusted global P value represents the probability of non-neutral selection based on the normalized frequency of non-synonymous mutations (i.e. missense, nonsense, essential splice site, and indel mutations). One gene reached significance after correction for multiple testing (q < 0.1, shown with an asterisk): *CDKN2A* in MDACC MBM. Left barchart shows the number of observed non-synonymous and synonymous mutations. Right and top barcharts are as described in Fig. 3.

Genes with a higher rate of somatic mutations than expected were also identified using dN/dS analyses (Fig. 6, Additional file 1). Top-ranking genes are more likely to be under non-neutral selection based on the normalized frequency of missense, nonsense, essential splice site, and indel mutations; however, just one gene reached significance after correction for multiple testing: *CDKN2A* in MDACC MBM. Top-ranking genes were largely distinct between MDACC and Duke MBM, and between MDACC brain and skin metastases. However, a few overlaps between MDACC brain and LN were detected: *CDKN2A*, *PPP2R1A*, and *KLRD1*.

**Fig. 7:**
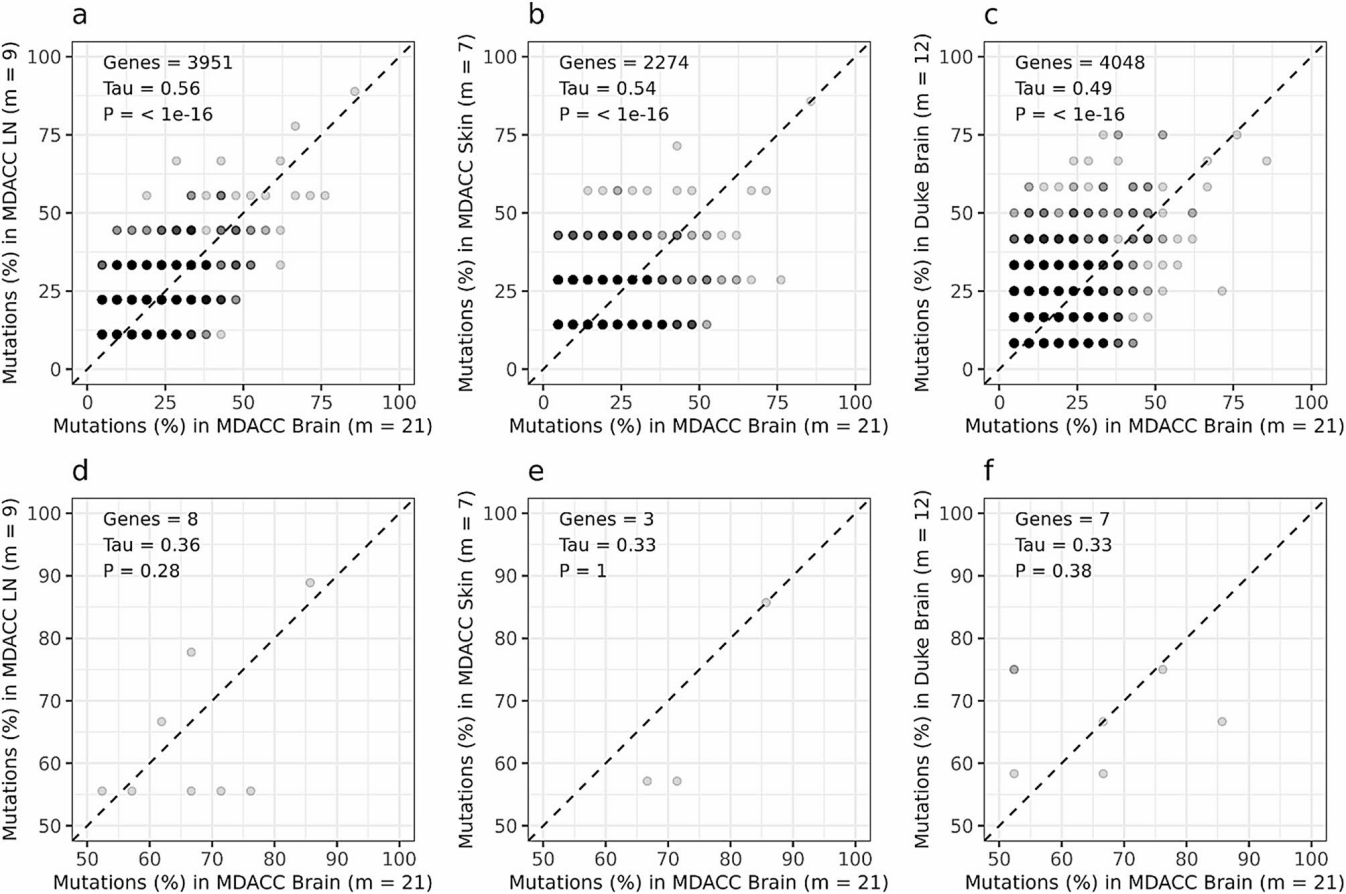
Shared mutations between cohorts and tumor locations. Shared mutations analysis comparing MDACC MBM (*n* = 17, m = 21) and Duke MBM (*n* = 12, m = 12), MDACC LN (*n* = 8, m = 9), and MDACC skin (*n* = 6, m = 7) metastases. Top row of panels: shared mutations analysis including all genes with non-synonymous, stop-loss, and stop-gain mutations, comparing MDACC MBM versus (**a**) MDACC LN, (**b**) MDACC skin metastases, and (**c**) Duke MBM. Bottom row of panels: shared mutations analysis using only genes with mutations present in > 50% of samples, comparing MDACC MBM versus (**d**) MDACC LN, (**e**) MDACC skin metastases, and (**f**) Duke MBM

Finally, we performed a rank-based test for concordance (Kendall’s Tau) to assess whether the ranks of gene mutation frequencies are statistically independent between MDACC MBM and Duke MBM, MDACC LN and skin metastases (Fig. 7). For tests that included all genes, the correlations between the rank of MDACC MBM gene mutations and any other sample set were statistically significant (Duke MBM: 4048 genes, Tau = 0.49, *p* < 1 × 10^–16^; MDACC LN: 3951 genes, Tau = 0.56, *p* < 1 × 10^–16^; MDACC skin: 2274 genes, Tau = 0.54, *p* < 1 × 10^–16^). When the analysis was repeated using the subset of genes with mutation frequencies > 50% in both sample sets, the correlations between the ranks of MDACC MBM and any other sample set were not statistically significant (Duke MBM: 7 genes, Tau = 0.33, *p* = 0.38; MDACC LN: 8 genes, Tau = 0.36, *p* = 0.28; MDACC skin: 3 genes, Tau = 0.33, *p* = 1).

### Comparison of gene expression in MBM versus LN and MBM versus skin metastases

Paired DGE and GSEA comparing patient-matched MBM versus LN metastases (22 MBM and 18 LN from 16 patients) and MBM versus skin metastases (12 MBM and 12 skin samples from 10 patients) were performed using RNAseq data from the MDACC cohort. Paired DGE identified 91 and 134 up-regulated genes with a log_2_ fold change > 1 and q-value < 0.05 in MBM relative to skin and LN metastases, respectively, 679 and 278 down-regulated genes with a log_2_ fold change <-1 and q-value < 0.05 in MBM relative to skin and LN metastases, respectively (Additional file 2).

**Fig. 8:**
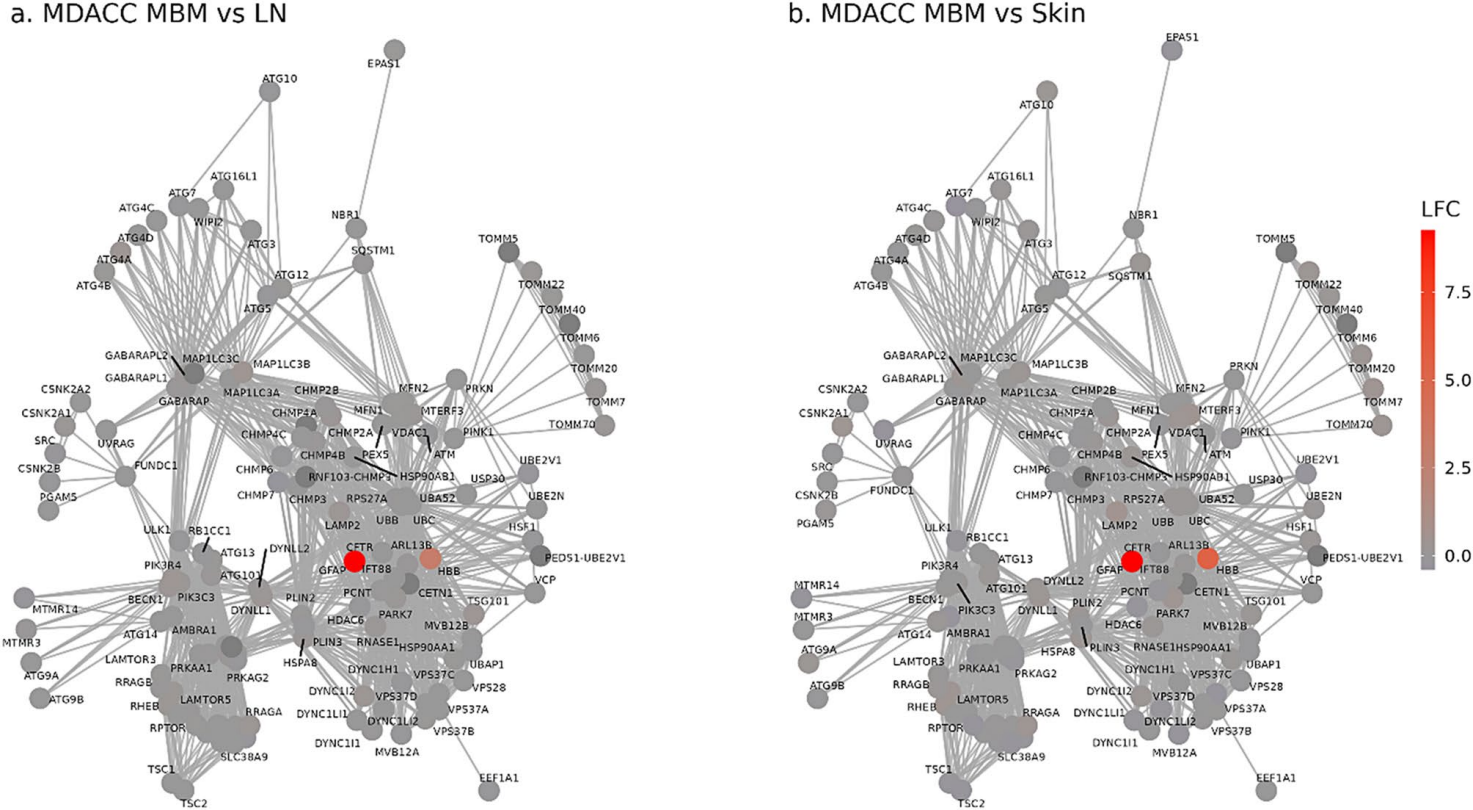
Autophagy pathway signaling in MBM. Network plots of log2 fold changes (LFC) in autophagy-related genes in (**a**) MDACC MBM versus LN metastases and (**b**) MDACC MBM versus Skin metastases. The most strongly upregulated genes were GFAP and HBB. Fold changes in other genes were low and did not reach significance

GSEA showed that autophagy signaling is upregulated in MBM compared to skin metastases (NES = 1.94, q = 0.018), with a similar trend observed in MBM compared to LN metastases (NES = 1.8, q = 0.1; Additional file 3). Based on this finding and previously published work describing a role for autophagy in tumor immune evasion [68], we investigated the individual genes that underpin the autophagy signaling pathway. Comparing both MBM versus LN and MBM versus skin metastases, the most strongly upregulated genes in MBM in autophagy pathways were glial fibrillary acidic protein (*GFAP*) and hemoglobin beta (*HBB*), whereas fold changes in most other autophagy-related genes were low and did not reach significance (q < 0.05) (Fig. 8). Log_2_ fold change in *GFAP* expression was 9.25 (q = 1.64 × 10^–14^) and 9.73 (q = 4.45 × 10^–43^) in MBM compared to skin and LN metastases, respectively, and *HBB* expression was 5.43 (q = 4.64 × 10^–21^) and 3.15 (q = 5.33 × 10^–10^), respectively (Additional file 2).

Based on comparison of DGE in ICr versus SQ implanted xenograft tumors in four different models (A375, MEWO, WM1361, and MEL624) [18], we identified significant up-regulation of the Autophagy pathway (R-HSA-9612973) in each cell line and across all cell lines (Table 2, Additional file 4: Supplementary Fig. 1). Autophagy genes *GFAP* and *HBB* did not underpin up-regulation of the pathway as these genes were significantly down-regulated in ICr relative to SQ in the majority of cell lines (MEWO, WM1361, and MEL624). *GFAP* and *HBB* were removed from the A375 cell line DGE analysis due to a sample with an extreme count outlier based on Cook’s distance.

**Table 2:** Autophagy pathway results in the xenograft dataset. Comparison between ICr and SQ implanted tumors in xenografts identified up-regulation of the autophagy pathway in ICr

Cell line	Pathway ID	Pathway Description	Set size	ES	NES	*P*
A375	R-HSA-9,612,973	Autophagy	123	0.56	2.09	1.00e-05
MEL624	R-HSA-9,612,973	Autophagy	145	0.20	21.37	1.00e-05
MEWO	R-HSA-9,612,973	Autophagy	145	0.35	34.74	1.00e-05
WM1361A	R-HSA-9,612,973	Autophagy	144	0.35	8.28	1.00e-05

Column names include: cell line; pathway ID, reactome pathway identifier; pathway description; set size, number of genes in the intersection of the pathway and dataset; ES, enrichment score; NES, normalized enrichment score; P, unadjusted permutation P value for pathway up-regulation

We analyzed DGE of the PCM samples among the stated contrasts, focusing on the Autophagy pathway (R-HSA-9612973). Significant up-regulation of the Autophagy pathway was not detected for any of the contrasts tested (Additional file 4: Supplementary Table 1).

### Immune cell infiltration in MBM

**Fig. 9:**
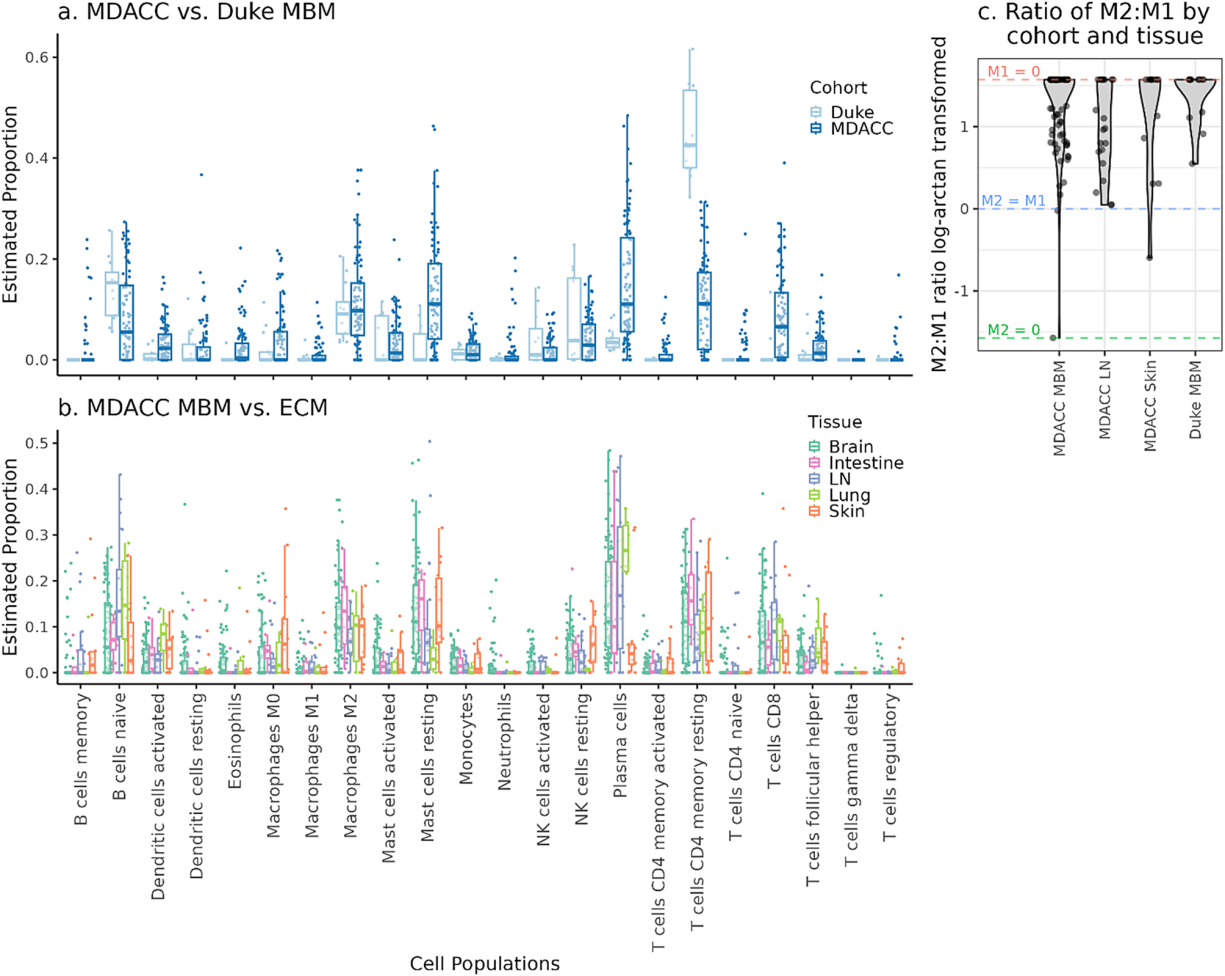
Tumor immune microenvironment. CIBERSORTx using mRNA data to estimate abundance of immune cells in (**a**) MDACC MBM (*n* = 17, m = 88) versus Duke MBM (*n* = 13, m = 13) and in (**b**) MDACC MBM versus ECM (Intestine *n* = 7, m = 8; LN *n* = 19, m = 22; Lung *n* = 5, m = 6; Skin *n* = 12, m = 14). (**c**) The ratio of M2:M1 macrophages by cohort and tissue (MDACC MBM, MDACC LN, MDACC Skin, Duke MBM). There was no significant difference detected in M2:M1 ratio comparing between MDACC MBM and any of the other sample sets. The M2:M1 ratio was transformed using atan (log(M2/M1)/ pi*2) to account for zeros and compositional data. Samples with zero M1 and zero M2 cells are not shown in panel c

To describe the tumor immune microenvironment of MBM compared to ECM, we performed immune cell profiling using CIBERSORTx, which uses gene expression data to estimate the relative abundance of 22 immune cell types. The relative abundance of most immune cell types was similar between the MDACC MBM (*n* = 74, m = 88) and Duke MBM (*n* = 13, m = 13) (Fig. 9a). However, a higher estimated relative abundance of resting CD4^+^ memory T cells was observed in Duke MBM compared to MDACC MBM. We also observed a higher estimated relative abundance of immune-suppressive M2 macrophages compared to tumor-suppressive M1 macrophages in both Duke MBM and MDACC MBM (Fig. 9a) and in MDACC ECM (Fig. 9b). To statistically test for these differences, we transformed M2:M1 ratios to account for zero values in the estimated M1 fractions (Fig. 9c). In total, 57/88 MBM in the MDACC dataset and 9/13 MBM in the Duke dataset had an estimated M1 fraction of zero. There were no significant differences detected in M2:M1 ratios comparing between MDACC MBM and any of the other sample sets (Duke MBM, MDACC LN, or MDACC skin metastases; Additional file 4: Supplementary Table 2).

**Fig. 10:**
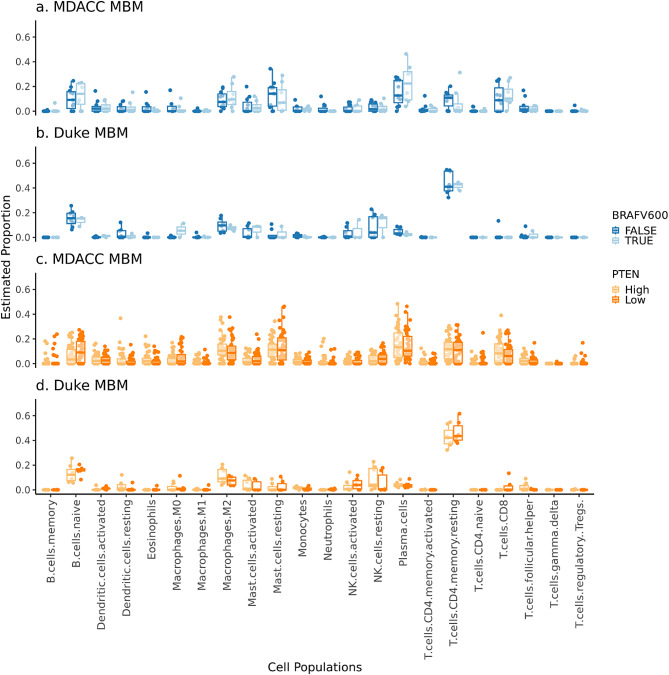
Tumor immune microenvironment by *BRAF* V600 mutation status and *PTEN* expression. CIBERSORTx using mRNA data to estimate abundance of immune cells in patients with and without a *BRAF * V600 mutation among (**a**) MDACC MBM (*n* = 15, m = 19) and (**b**) Duke MBM (*n* = 11, m = 11); and in patients with high versus low *PTEN* expression among (**c**) MDACC MBM (*n* = 74, m = 88) and (**d**) Duke MBM (*n* = 13, m = 13)

Next, based on previously published work demonstrating alterations in the immune microenvironment based on *BRAF* V600E mutation status [64], we investigated differences in immune cell fractions in MBM based on the presence of the *BRAF* V600 mutation and high vs. low *PTEN* expression in both MDACC and Duke cohorts. Immune cell fractions were globally similar in MBM with and without *BRAF* V600 mutations in both cohorts (Fig. 10a). Similarly, when comparing MBM with high vs. low *PTEN* expression, cell fractions were similar in both cohorts (Fig. 10b).

### Survival analysis

**Fig. 11:**
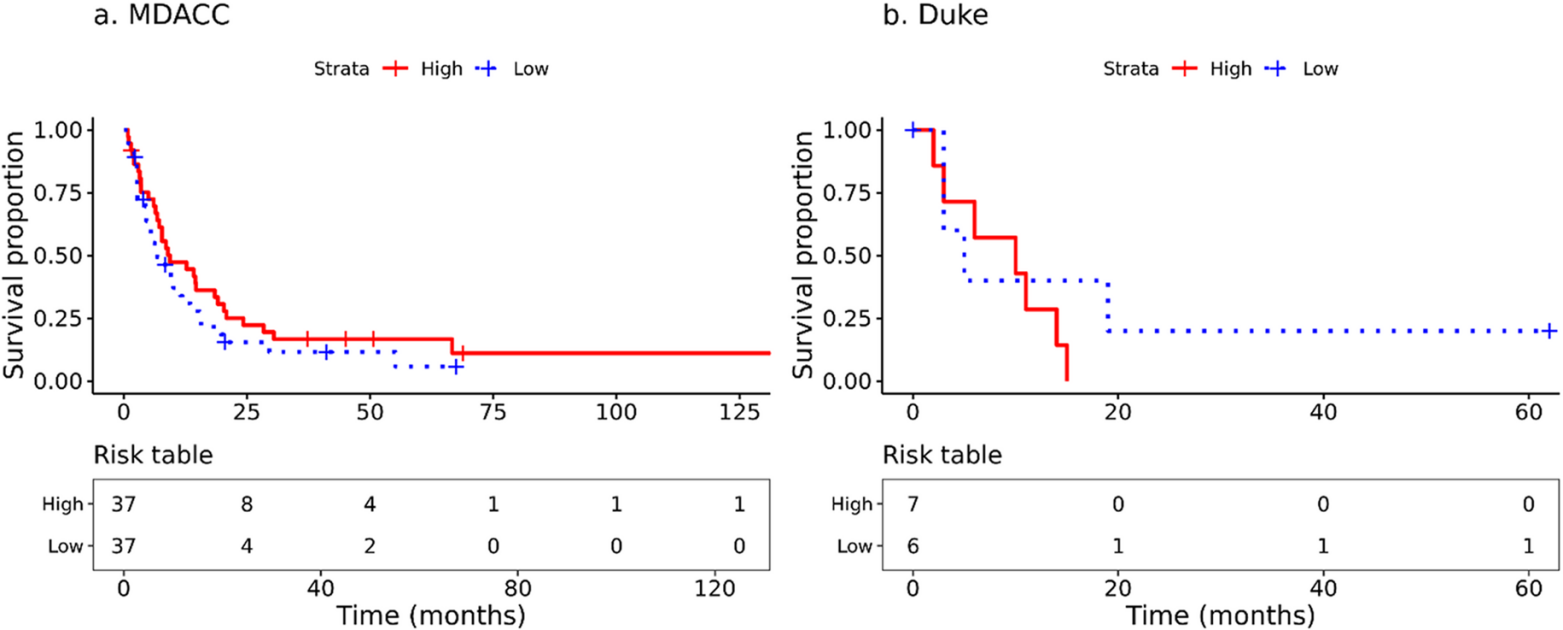
Overall survival by CD8 + T cell fraction. Kaplain-Meier OS from craniotomy comparing between CD8 + T cell high versus low (split on median) in MBM from the (**a**) MDACC (*p* = 0.010) and (**b**) Duke (*p* = 0.50) datasets. Results in (**a**) have been previously published [18]

In the MDACC analysis cohort, seven patients had survival data from multiple accession times. Time to death or last follow-up for these patients was calculated from the earliest craniotomy. Three patients had multiple samples extracted on the only or earliest craniotomy event reported. For these patients, one sample was randomly selected. To evaluate the effect of infiltrating immune cell fractions (e.g., M1 and M2 macrophages, CD4 + and CD8 + T cells) on OS from craniotomy, we used log-transformed cell fractions as predictors. A small constant (Duke: c = 0.00107, MDACC: c = 0.000473) representing half of the minimum non-zero cell fraction was added to each cell type fraction before their transformation to avoid taking logs of zeros. Similar to the previously published analysis, we found that increased CD8^+^ T cell fractions were associated with OS from craniotomy in the MDACC MBM (95% CI 0.805–0.972, *p* = 0.01) (Fig. 11a). There was no significant association detected between OS from craniotomy and immune cell fraction in the other cell types that were analyzed (CD4^+^ T cells, M1 or M2 macrophages). We then investigated whether CD8^+^ T cell fraction was associated with OS from craniotomy in the Duke cohort. No statistically significant difference in OS was observed (*p* = 0.50) (Fig. 11b), which may reflect the small sample size (*n* = 13).

**Fig. 12:**
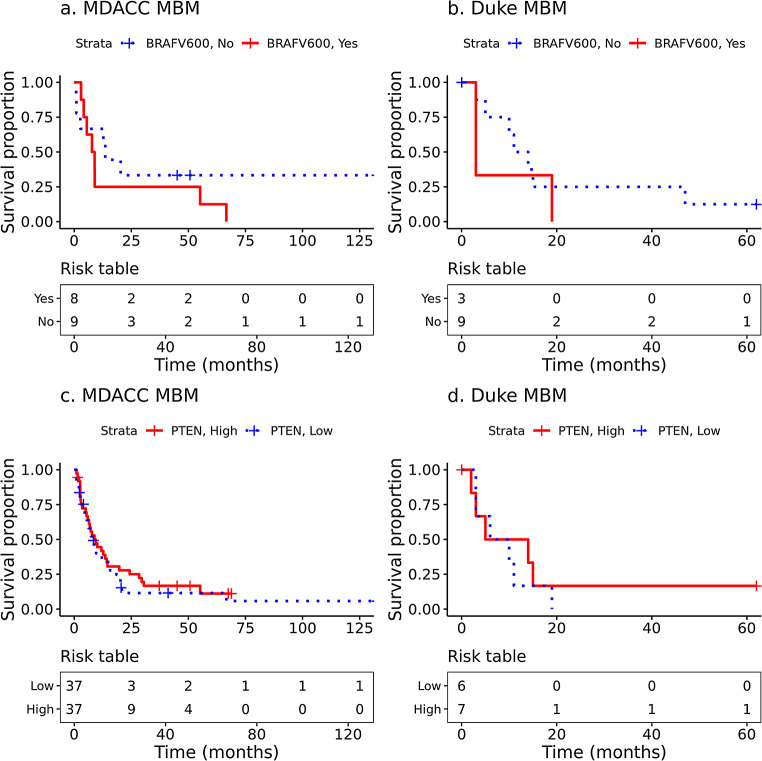
Overall survival by *BRAF*V600 mutation status or PTEN expression. Kaplain-Meier OS from craniotomy comparing between *BRAF* V600 mutation status and its association with OS from craniotomy in (**a**) MDACC MBM (*p* = 0.359) and (**b**) Duke MBM (*p* = 0.368). Kaplain-Meier OS from craniotomy comparing between *PTEN* expression high versus low (split on median) and its association with OS from craniotomy in (**c**) MDACC MBM (*p* = 0.491) and (**d**) Duke MBM (*p* = 0.602)

Finally, we investigated whether there was an association between *BRAF* V600 mutation or *PTEN* expression level with OS from craniotomy. In both the MDACC and Duke cohorts, there was no statistically significant difference detected in OS from craniotomy when comparing between *BRAF* V600 mutation present versus absent (Fig. 12a and b) or *PTEN* high (> median) versus low (< median) expression (Fig. 12c and d).

## Discussion

Brain metastases remain a common cause of morbidity and mortality in patients with advanced melanoma. Understanding the genetic and molecular features and immune microenvironment of MBM will facilitate the design of clinical trials for this patient population. In our expanded analyses of the largest published cohort of patient-matched MBM and ECM, we characterized the genetic and immune landscape of MBM and ECM and reported findings in an independent dataset. Key strengths of this study include the large sample size and availability of patient-matched samples in the previously published MDACC dataset in addition to the availability of RNA, DNA, and clinical data from patients in both the MDACC and the independent Duke datasets.

We observed concordance both in genes with high frequencies of somatic mutations in brain, lymph node, and skin metastases and in mutation rates of clinically relevant genes in the MDACC and independent Duke cohorts. Specifically, *BRAF*, *NRAS*, and *PTEN* mutations were frequent in both brain metastasis cohorts. This finding is consistent with the previously published analysis of this dataset [18] and with other studies showing a similar mutational landscape between MBM and ECM [10, 22, 28]. Similarly, *TTN* was among the most frequently somatically mutated genes in both cohorts regardless of tissue site (brain, LN, or skin), and *MUC16* was frequently mutated in both MDACC and Duke MBM, similar to previous reports in primary cutaneous melanomas [34, 38]. While *BRAF*, *NRAS*, and *PTEN* mutations are known to be clinically relevant given targeted therapies, the clinical utility of other identified mutations requires further investigation. *TTN* mutations have been suggested as markers of better prognosis and of response to immune checkpoint inhibitors in solid tumors [32, 65], though the large size of the *TTN* gene itself may confound such analyses.

To address the possibility that the large size of *TTN* confounded our analyses, we also examined genes with a high frequency of somatic alterations based on dN/dS analysis and found that one gene reached significance after correction, which was *CDKN2A* in the MDACC MBM. *CDKN2A* is a tumor suppressor gene, and the *CDKN2A* locus is commonly lost in the development of invasive melanoma and melanoma metastases [72]. A prior meta-analysis of molecular alterations in MBM reported a high rate of single nucleotide variants and gene copy number variations in *CDKN2A/B* in MBM [51]. In addition, a retrospective, single-center study of resected brain metastases across tumor types identified high rates of *CDKN2A* mutation and *CDKN2A/B* co-deletion in brain metastases from multiple tumor types. *CDKN2A/B* co-deletion was associated with increased risk of CNS recurrence after resection [48]. *CDKN2A/B* encode p16 and 15, which interact with CDK4 and CDK6, which can be targeted using the CKD4/6 inhibitors ribociclib, palbociclib, and abemaciclib [48].

Our study suggests that the autophagy signaling pathway may be upregulated in MBM compared to ECM. The role of autophagy in melanoma development and metastasis remains incompletely defined, though a number of studies suggest autophagy promotes melanomagenesis through a variety of mechanisms [52]. Autophagy has been shown to play a role in multiple mechanisms of tumor immune evasion, including avoidance of destruction by cytotoxic T cells, degradation of cytotoxic granules such as granzyme B and perforin, and reduction of immune recognition of tumor antigens [9]. These roles of autophagy can promote a TME that favors tumor growth and immune evasion. In addition, preclinical studies have demonstrated that autophagy can contribute to resistance to targeted therapy in models of *BRAF*-mutant melanoma [50]. A growing body of literature has demonstrated that dysregulation of autophagy contributes to melanomagenesis and progression of *BRAF* V600-mutant melanoma [19]. Autophagy has been found to contribute to acquired resistance to targeted therapies in *BRAF* V600-mutant melanoma [39], which has led to interest in targeting autophagy pathways as a strategy to improve outcomes in *BRAF *V600-mutant melanoma. These findings have led to multiple clinical trials investigating the therapeutic potential of manipulating autophagy pathways in patients with *BRAF* V600-mutant melanoma. Early approaches investigated the activity of combined chemotherapy and autophagy inhibition, such as temsirolimus and hydroxychloroquine, an autophagy inhibitor, in patients with advanced solid tumors, including melanoma [54]. A phase I/II trial of dabrafenib + trametinib + hydroxychloroquine in patients with *BRAF*-mutant melanoma demonstrated promising response rates in a treatment refractory patient population, but did not meet the pre-specified 1 year PFS rate of 60% [47]. A randomized trial comparing dabrafenib + trametinib in combination with either hydroxychloroquine or placebo in patients with *BRAF* V600 mutant melanoma (NCT04527549) terminated early due to slow accrual.

We observed that the autophagy pathway may be upregulated in MBM compared to both LN and skin metastases. On the individual-gene level in patient MBM, this finding seemed to be driven by upregulation of *GFAP* and *HBB*. Similar to our observation of upregulation of *HBB* in patient MBM samples, a multi-omic analysis of a large, multi-institutional melanoma cohort identified significant upregulation of *HBB* in addition to hemoglobin subunit alpha 1 mRNA levels in MBM compared to primary cutaneous melanomas [29]. The upregulation of *GFAP* that we observed in the patient MBM samples could indicate that our results were driven by the presence of surrounding reactive astrocytes/glia and blood cells in the craniotomy specimens. In addition, circulating GFAP levels have been shown to correlate with severity of intracranial pathologies such as traumatic brain injury [1]. In one study, patients with glioblastoma multiforme and high levels of circulating GFAP + monocytes had a worse prognosis [62]. Although the use of circulating GFAP levels for diagnosis and prognosis of brain metastasis requires further study prior to incorporation into routine clinical practice, high circulating GFAP levels may serve as a biomarker of brain injury. In our study, high GFAP expression may be related to brain injury in the setting of brain metastasis and craniotomy. Brain metastases have been shown to have low levels of GFAP expression except when contaminated by surrounding brain tissue [31]; therefore, our observation could indicate contamination of brain metastasis specimens by surrounding brain tissue.

The observed changes in GFAP expression could alternatively be due to neuronal-like differentiation within melanoma tumors, which may be increased in MBM. A previous analysis of multiple cancer cell lines, including A375 cells, compared expression of neural markers when the cells were grown in culture vs. SQ xenografts [70]. Compared to A375 cells grown in culture, A375 SQ xenograft tumors demonstrated up-regulation of glial and neuronal genes, including *GFAP.* Another study using primary nodular melanoma specimens reported a pattern of neural differentiation in which cells abutting the basement membrane acquired GFAP positivity [30]. A recent study which used multi-modal single-cell transcriptomics to characterize treatment-naïve MBM and ECM described a pattern of neuronal-like (albeit not astrocyte-like) differentiation in MBM compared to ECM [3]. In addition, a gene expression profiling study of patient-matched MBM and ECM found that MBM demonstrate a brain-like gene expression profile compared to ECM, with increased expression of genes involved in synaptic processes, brain-specific cell development, and neuronal processes [35].

To assess the role of the autophagy pathway with respect to clinical outcome in metastatic melanoma, we analyzed data from an independent cohort of patients with primary cutaneous melanoma (PCM). Comparisons between PCMs that recurred in the brain or elsewhere versus those who did not recur were inconclusive. When we examined expression of the same autophagy pathways in preclinical xenograft models of melanoma, we found increased autophagy pathway expression in ICr versus SQ tumors across four different xenografts, a finding which was not driven by *GFAP* expression. However, within each cell line, there are expected to be fundamental differences between ICr and SQ cells related to the different organ sites, which could lead to differential expression of a significant number of pathways, including but not limited to autophagy signaling pathways. We observed opposite direction of effect sizes regarding *GFAP* and *HBB* expression in human versus xenograft specimens. *GFAP* and *HBB* were up-regulated in human MBM compared to ECM specimens but conversely down-regulated in ICr compared to SQ xenografts.

Our results suggest that autophagy signaling pathways may be up-regulated in MBM compared to ECM. However, there are significant limitations to this interpretation of our results. Our observation of up-regulated autophagy signaling in human MBM specimens was likely driven by *GFAP* expression from surrounding brain parenchyma given the observation of reduced *GFAP* expression in ICr versus SQ xenograft tumors previously discussed. Further, our observation of increased autophagy signaling in ICr xenografts is of unclear biologic significance. The sample size in the xenograft analysis was small, and the results may be sensitive to technical variation in the xenograft samples (Additional file 4: Supplementary Fig. 2). The biologic and therapeutic importance of our autophagy findings remains unknown and requires further study. On going and future studies using single-cell RNAseq and spatial transcriptomic technology will help dissect the biologic changes in melanoma cells versus the TME.

In our analysis of immune cell subpopulations, we observed an increased proportion of immune-suppressive M2 macrophages compared to tumor-suppressive M1 macrophages in both MBM and ECM. There were no differences in M2:M1 ratio comparing between MDACC MBM and any other sample set. We did not observe an association between M1 or M2 macrophage cell fraction and survival, similar to previous reports in melanoma [34]. Tumor associated macrophages (TAMs) are a heterogeneous cell population which exhibits plasticity to respond to the TME and therefore can exert complex effects on tumor growth [42]. Single-cell transcriptomic studies of treatment-naïve MBM and ECM described two major macrophage clusters [3]. Although these macrophage clusters did not align with the classic M1- and M2-like phenotypes, they represented distinct classes of macrophages, either with expression of genes associated with anti-tumor immunity or pro-tumorigenic effect, and MBM were enriched for pro-tumorigenic macrophages, suggesting that macrophage polarization is an important feature of the TME. Similarly, another scRNAseq study of MBM and melanoma leptomeningeal and skin metastases identified high levels of immunosuppressive myeloid cell populations in MBM [58]. Additional work is needed to clarify the role of macrophage polarization in the brain microenvironment and development of the brain metastatic niche.

This study has several limitations. First, there was a difference in tissue processing techniques between the two institutions. Whereas MDACC MBM and ECM and Duke ECM were stored as FFPE tissue blocks, the Duke MBM were frozen. Differences in tissue processing may have limited the reliability of comparing between the two MBM cohorts. In addition, the MDACC analysis used mRNAseq, whereas the Duke cohort used Total RNAseq. Obtaining RNAseq data on the matched ECM from the Duke dataset was not feasible due to tissue degradation. Prior studies demonstrating unique, potentially targetable features in patient-matched brain metastases compared to ECM highlight the importance and clinical relevance of comparing matched tissue from the same patients [5, 18]; however, availability of patient-matched MBM and ECM is limited in the current literature. Although the MDACC dataset represents the largest cohort of published patient-matched MBM and ECM, the sample size of our independent Duke dataset was small. Our sample size was too small to describe the effect of systemic therapies on the TME or gene expression profiles. Further, it is important to note that a minority of patients in both datasets received modern systemic therapies such as checkpoint inhibitors, which demonstrate a significant survival advantage compared to historic controls.

## Conclusions

This study demonstrates that the landscape of genetic alterations is similar between MBM and ECM and identifies possible targets for future studies of the pathogenesis of MBM. Autophagy signaling pathways may be upregulated in MBM compared to ECM; however, this finding was driven by upregulation of *GFAP* and *HBB* and may reflect reactive astrocytes in the TME. In addition, we describe an increased proportion of immune suppressive M2 compared to tumor suppressive M1 macrophages in both MBM and ECM, which was not prognostic, but which may contribute to an immune suppressive microenvironment. Future work to more fully characterize the immune landscape of MBM will be important for developing optimal treatment strategies.

## Electronic supplementary material

Below is the link to the electronic supplementary material.

Supplementary Material 1

Supplementary Material 2

Supplementary Material 3

Supplementary Material 4

## Data Availability

The genomic and phenotypic data from the Duke cohort were deposited into the NCBI’s dbGaP repository (accession no. phs003009.v1.p1). Inferential analyses were carried out using the R Statistical Environment [53] along with extension packages from the Comprehensive R Archive Network (CRAN; https://cran.r-project.org/), including tidyverse [66] and the Bioconductor project [27]. These analyses were carried out with adherence to the principles of reproducible analysis using the knitr package [69] for generation of dynamic reports and Duke’s gitlab (https://gitlab.oit.duke.edu/) for source code management. The code for replicating the statistical analyses was made available through a public source code repository (https://gitlab.oit.duke.edu/dcibioinformatics/pubs/anders-kennedy-mbm).

## Abbreviations

BTBR: Brain Tumor Biorepository
CNS: Central Nervous System
DGE: Differential Gene Expression
ECM: Extracranial Metastases
FFPE: Formalin-Fixed Paraffin-Embedded
GFAP: Glial Fibrillary Acidic Protein
GSEA: Gene Set Enrichment Analysis
HBB: Hemoglobin Beta
ICr: Intracranial
IRB: Institutional Review Board
LN: Lymph Node
MBM: Melanoma Brain Metastases
MDACC: MD Anderson Cancer Center
NK: Natural Killer
OS: Overall Survival
OXPHOS: Oxidative Phosphorylation
PCM: Primary Cutaneous Melanoma
(sc)RNAseq: (Single Cell) RNA Sequencing
SRS: Stereotactic Radiosurgery
SQ: Subcutaneous
TME: Tumor Microenvironment
TTN: Titin
WBRT: Whole Brain Radiation Therapy
WES: Whole Exome Sequencing
