# Supplementary material for: A comprehensive, multi-center, immunogenomic analysis of melanoma brain metastases
Source: Acta Neuropathol Commun. 2025 Jun 2;13:123. doi: 10.1186/s40478-025-02035-7 (PMC12128383; doi:10.1186/s40478-025-02035-7)
Supplement: Supplementary file 4 — Supplementary Material 4 [file 40478_2025_2035_MOESM4_ESM.docx]

**Supplementary Tables**

**Supplementary Table 1. Autophagy pathway results in the PCM dataset.** Autophagy pathway results based on comparison between PCMs with different recurrence types. Column names include: Contrast; Pathway ID, Reactome pathway identifier; Pathway Description; Set size, number of genes in the intersection of the pathway and dataset; ES, enrichment score; NES, normalized enrichment score; P, unadjusted p value for pathway up-regulation

| **Contrast** | **Pathway ID** | **Pathway Description** | **Set size** | **ES** | **NES** | **P** |
| --- | --- | --- | --- | --- | --- | --- |
| PCM with any recurrence versus no recurrence | R-HSA-9612973 | Autophagy | 144 | 0.30 | 0.41 | 0.85 |
| PCM with ECM recurrence versus no recurrence | R-HSA-9612973 | Autophagy | 141 | 0.30 | 0.39 | 0.86 |
| PCM with brain recurrence versus no recurrence | R-HSA-9612973 | Autophagy | 147 | 0.30 | 0.42 | 0.93 |
| PCM with brain recurrence versus ECM recurrence | R-HSA-9612973 | Autophagy | 138 | 0.41 | 0.68 | 0.79 |

**Supplementary Table 2. Pairwise Wilcoxon test results for comparing macrophage ratios between sample groups.** Column names include: Comparison, description of sample group comparison; Estimate (95% CI), estimate of the difference of the location parameter between groups with upper and lower 95% confidence intervals; Adjusted p-value, Bonferroni adjusted p-value. Abbreviations include: LN = lymph node. MBM = melanoma brain metastases. MDACC = MD Anderson Cancer Center.

| **Comparison** | **Estimate (95% CI)** | **Adjusted p-value** |
| --- | --- | --- |
| MDACC MBM-MDACC LN | 0.095 (4e-05, 0.56) | 0.23 |
| MDACC MBM-MDACC Skin | 3.1e-05 (-5.8e-06, 7.1e-06) | 1 |
| MDACC MBM-Duke MBM | -1.8e-06 (-0.12, 4.9e-07) | 1 |
| MDACC LN-MDACC Skin | -6e-05 (-0.61, 4.5e-05) | 1 |
| MDACC LN-Duke MBM | -0.37 (-0.77, 5.3e-05) | 0.27 |
| MDACC Skin-Duke MBM | -4.5e-05 (-0.44, 4.1e-05) | 1 |

**Supplementary Figures**

**Supplementary Figure 1**


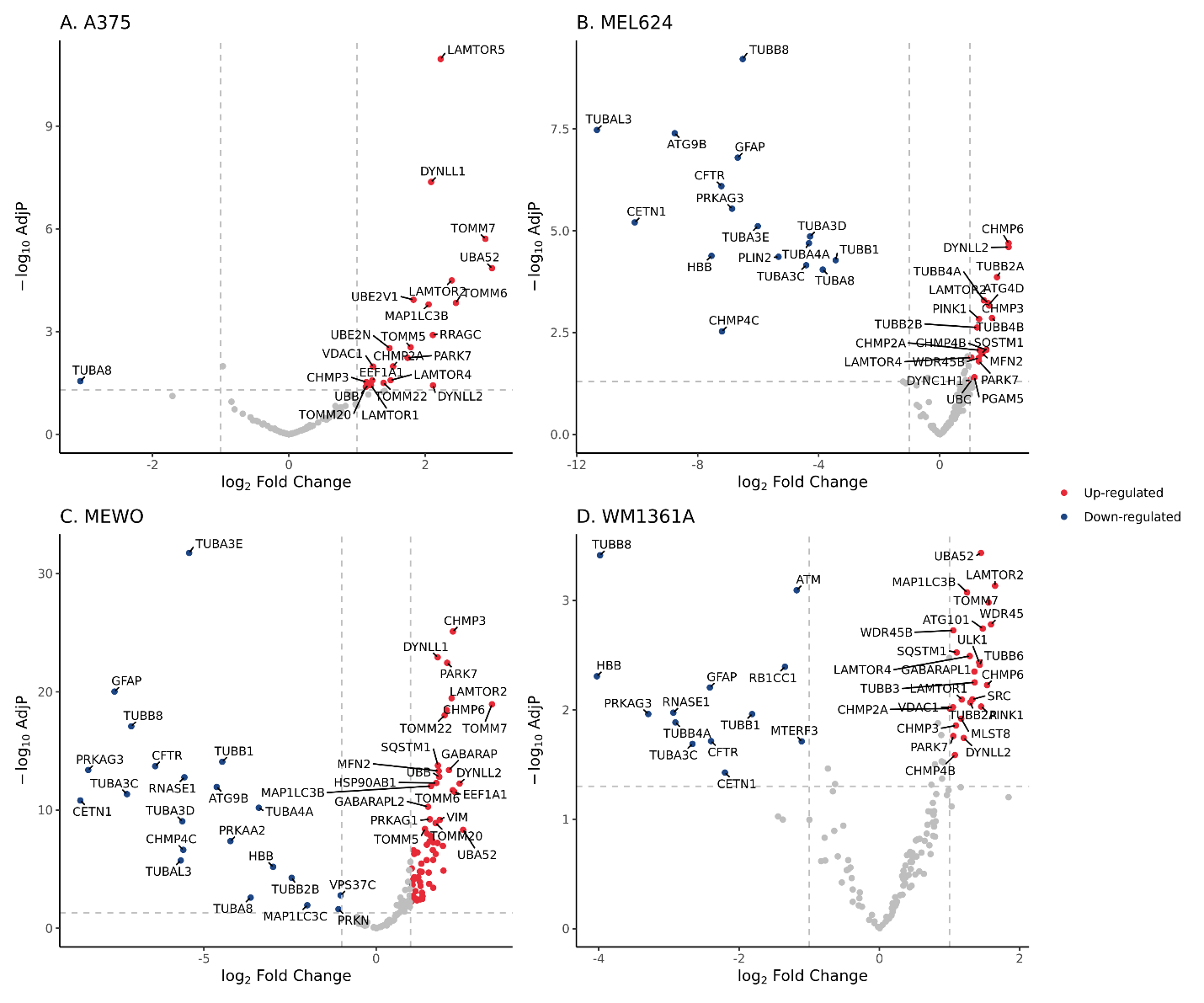


**Supplementary Figure 1**. **Volcano plots of differentially expressed Autophagy pathway genes in ICr versus SQ within xenograft cell lines.** Red and blue dots represent significantly (-log10(AdjP) < 0.05) up-regulated (log2FoldChange > 1) and down-regulated (log2FoldChange < -1) genes in the Autophagy pathway, respectively, in intracranial (ICr) compared to subcutaneous (SQ) tumors. The y-axis depicts -log10 adjusted p-values.

**Supplementary Figure 2**


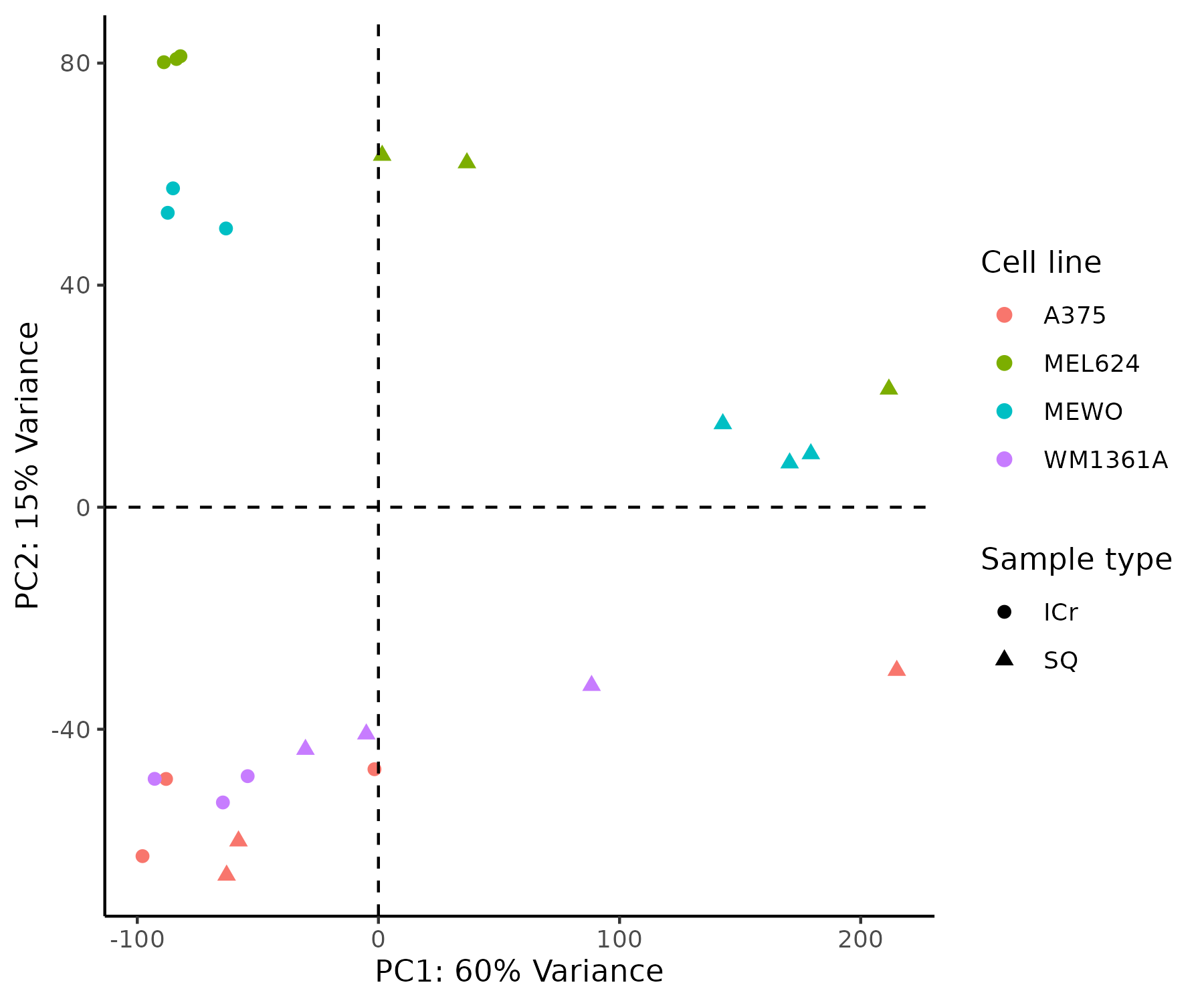


**Supplementary Figure 2**. **Principal component analysis of gene expression from the analyzed xenograft samples.** Sample points are colored by cell line (A375, MEL624, MEWO, or WM1361A) with the shape based on sample type (intracranial (ICr) or subcutaneous (SQ)).
